# Rapid Induction of Multifunctional Antibodies in Rabbits and Macaques by Clade C HIV-1 CAP257 Envelopes Circulating During Epitope-Specific Neutralization Breadth Development

**DOI:** 10.3389/fimmu.2020.00984

**Published:** 2020-06-02

**Authors:** Delphine C. Malherbe, Constantinos Kurt Wibmer, Molati Nonyane, Jason Reed, D. Noah Sather, David A. Spencer, Jason T. Schuman, Biwei Guo, Shilpi Pandey, Harlan Robins, Byung Park, Deborah H. Fuller, Jonah B. Sacha, Penny L. Moore, Ann J. Hessell, Nancy L. Haigwood

**Affiliations:** ^1^Oregon National Primate Research Center, Oregon Health and Science University, Beaverton, OR, United States; ^2^Centre for HIV and STIs, National Institute for Communicable Diseases, National Health Laboratory Service, Johannesburg, South Africa; ^3^Vaccine and Gene Therapy Institute, Oregon Health and Science University, Beaverton, OR, United States; ^4^Center for Global Infectious Disease Center, Seattle Children's Hospital Research Foundation, Seattle, WA, United States; ^5^Bruker Daltonics, Portland, OR, United States; ^6^Fred Hutchinson Cancer Research Center, Seattle, WA, United States; ^7^Biostatistics Unit, Primate Genetic Program Oregon National Primate Research Center, Oregon Health and Science University, Beaverton, OR, United States; ^8^AIDS Division, Department of Microbiology, Washington National Primate Research Center, University of Washington, Seattle, WA, United States; ^9^Antibody Immunity Research Unit, Faculty of Health Sciences, University of the Witwatersrand, Johannesburg, South Africa; ^10^Centre for the AIDS Programme of Research in South Africa (CAPRISA), University of KwaZulu-Natal, Durban, South Africa; ^11^Division of Medical Virology, Department of Pathology, Institute of Infectious Diseases and Molecular Medicine, University of Cape Town, Cape Town, South Africa; ^12^Molecular Microbiology and Immunology, School of Medicine, Oregon Health and Science University, Portland, OR, United States

**Keywords:** HIV vaccine, envelope immunogen, rabbit, NHP, neutralizing antibodies, co-immunization, ADCC, T_FH_ responses

## Abstract

We report here on HIV-1 immunization results in rabbits and macaques co-immunized with clade C gp160 DNA and gp140 trimeric envelope vaccines, a strategy similar to a recent clinical trial that showed improved speed and magnitude of humoral responses. Clade C envelopes were isolated from CAP257, an individual who developed a unique temporal pattern of neutralization breadth development, comprising three separate “Waves” targeting distinct Env epitopes and different HIV clades. We used phylogeny and neutralization criteria to down-select envelope vaccine candidates, and confirmed antigenicity of our antigens by interaction with well-characterized broadly neutralizing monoclonal antibodies. Using these envelopes, we performed rabbit studies that screened for immunogenicity of CAP257 Envs from timepoints preceding peak neutralization breadth in each Wave. Selected CAP257 envelopes from Waves 1 and 2, during the first 2 years of infection that were highly immunogenic in rabbits were then tested in macaques. We found that in rabbits and macaques, co-immunization of DNA, and protein envelope-based vaccines induced maximum binding and neutralizing antibody titers with three immunizations. No further benefit was obtained with additional immunizations. The vaccine strategies recapitulated the Wave-specific epitope targeting observed in the CAP257 participant, and elicited Tier 1A, 1B, and Tier 2 heterologous neutralization. CAP257 envelope immunogens also induced the development of ADCC and T_FH_ responses in macaques, and these responses positively correlated with heterologous neutralization. Together, the results from two animal models in this study have implications for identifying effective vaccine immunogens. We used a multi-step strategy to (1) select an Env donor with well-characterized neutralization breadth development; (2) study Env phylogeny for potential immunogens circulating near peak breadth timepoints during the first 2 years of infection; (3) test down-selected Envs for antigenicity; (4) screen down-selected Envs in an effective vaccine regimen in rabbits; and (5) advance the most immunogenic Envs to NHP studies. The results were an induction of high titers of HIV-1 envelope-specific antibodies with increasing avidity and cross-clade neutralizing antibodies with effector functions that together may improve the potential for protection in a pre-clinical SHIV model.

## Introduction

One of the greatest challenges in the HIV vaccine field remains the design of immunogens that elicit neutralizing antibodies (NAbs) able to tackle the worldwide diversity of HIV isolates. HIV clade C accounts for more than half of infections worldwide and is the predominant subtype affecting sub-Saharan African populations (1), making that part of the world the area most desperately in need of a vaccine. Protection from infection requires the generation of antibodies directed to the envelope (Env) glycoprotein (2) and heterologous NAbs develop in about half of chronically infected individuals (3), with a subset of individuals developing broadly neutralizing antibodies (bNAbs) (4). However, despite over 20 years of research and many different immunogen designs, bNAbs have proven impossible to elicit by vaccination (5) because antibodies with the potential to target sites of vulnerability on Env are rare and are often negatively selected by the immune system (6, 7). Thus, increasing the efficacy of HIV vaccines remains a critical goal. Different approaches have been taken to improve vaccine design such as engineering new immunogens based on native-like gp140 trimers (8, 9) or modifications to improve interactions with germline B cell receptors (10–12). We have focused on developing HIV immunogens derived from natural envelope quasispecies that evolved in subjects with neutralization breadth (13, 14).

Several comprehensive longitudinal studies of individuals who develop potent bNAbs during natural infection have provided evidence of how this process is accomplished during infection (4, 15–18) and have informed immunogen design. It was recently shown that HIV infected subjects can develop bNAb specificities to multiple epitopes and/or bNAb development can depend on multiple lineages (6, 16, 17, 19–22). CAP257, a clade C infected individual and the source of the Env immunogens in this study, sequentially developed bNAbs directed to three distinct targets on HIV-1 Env (15). Therefore, the goal of the current work was to use a clade C infected subject who developed NAb breadth as a source of immunogens to test whether our approach, developed with clade B envelopes, using envelopes selected at specific timepoints during the development of neutralization breadth—including very early after infection—could be expanded to another Env subtype. In addition, a very early (week 7) env from CAP257 was available with some characteristics of Transmitted/Founder (T/F) envelopes that differentiate them from chronic viruses in particular in clades A, C, and D featuring shorter variable loops and fewer PNGs (23–25). Although controversial, it has been proposed that T/F envelopes would be better immunogens than envelopes from chronic viruses (16) or that they could “jump start” the process to elicit bNAbs (26). The comparative studies in rabbits and macaques provide insights into the relative potency and specificity of humoral responses to guide the selection of vaccines for testing in challenge studies.

## Results

### Source of Lineage-Based Envelope Clones and Characterization of Env Quasispecies

CAP257 is a participant of the CAPRISA 002 Acute Infection Cohort, a prospective cohort of high risk HIV seronegative women established in South Africa in 2004. In this cohort, HIV-negative individuals have documented times of seroconversion, making the timing, and clinical features of infection well-known. CAP257 developed broad NAbs 3 years post-seroconversion, neutralizing 82% of tested viruses (27). Longitudinal mapping revealed that neutralization breadth in CAP257 was not due to the targeting of a single epitope but, instead was due to the sequential development of three distinct epitope specificities over a period of 4.5 years post-infection (ypi) (15). These three specificities, termed neutralization Waves, sequentially targeted the V2 region [1st NAb breadth peak at 67 weeks post-infection (wpi)], then the CD4 binding site (2nd NAb breadth peak at 122 wpi), and finally a quaternary epitope that remains undefined (3rd NAb breadth peak at 213 wpi).

**Figure 1:**
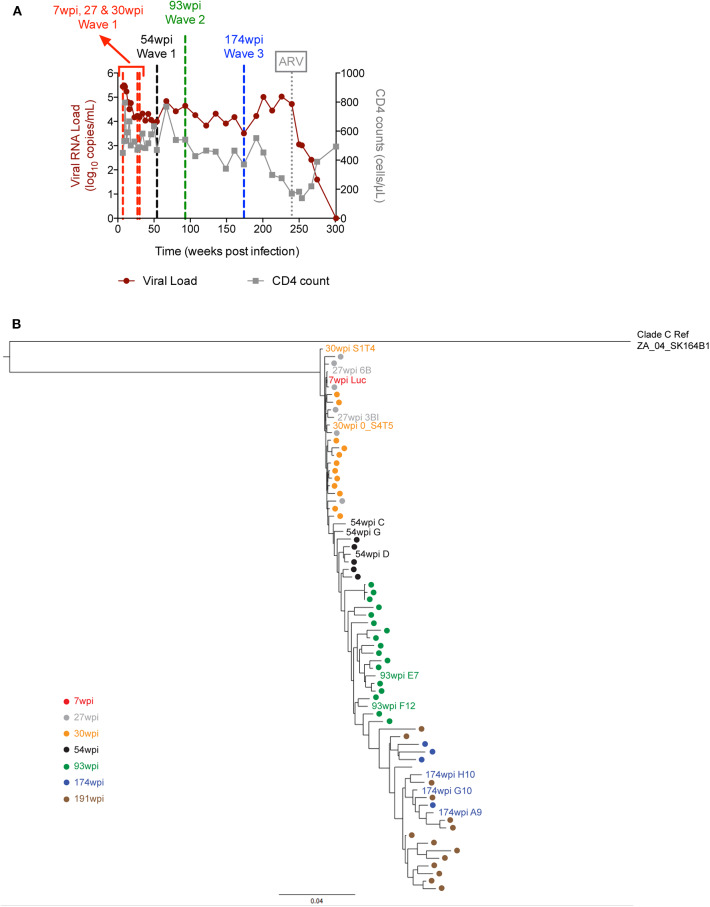
Plasma viral load, CD4 counts and envelope phylogenetic tree in CAP257. **(A)** HIV plasma viral load was determined over the course of infection and is expressed as log10 viral copies per ml of plasma for subject CAP257. CD4 T cell counts were determined longitudinally and are expressed as CD4 positive cells per microliter of blood. The vertical dashed line at week 240 indicates the start of ARV treatment and the other vertical lines indicate the time points from which vaccine envelopes were selected. **(B)** CAP257 maximum-likelihood (ML) phylogenetic tree. CAP257 envelopes were generated by single genome amplification (15). Sequences of 71 functional clones were aligned with HIVAlign (https://www.hiv.lanl.gov/content/sequence/VIRALIGN/viralign.html) to clade C reference ZA_04_SK164B1 in order to generate the ML tree rooted to ZA_04_SK164B1 with the DIVEIN program (https://www.indra.mullins.microbiol.washington.edu/DIVEIN/) (28).

The time-course of the plasma viral load (pVL) and CD4 counts over a period of 300 weeks is displayed in Figure 1A. When the CD4 counts dropped below 200 cells per microliter at week 240 (4.6 ypi), CAP257 began anti-retroviral (ARV) treatment (27), and the pVL quickly decreased to undetectable levels. Before that timepoint, the plasma viral load ranged from the peak value of 306,000 copies/mL (9 wpi) down to 3,220 copies/mL (174 wpi). We cloned a total of 71 full-length *env* genes from CAP257 plasma obtained at seven longitudinal timepoints spanning 3.7 years from 7 to 191 wpi. The maximum phylogenetic tree (Figure 1B) shows the evolution of *env* divergence and diversity over time in CAP257 quasispecies. The envelope diversity was compared to the 7wpi_Luc clone in a Highlighter analysis to illustrate the accumulation of synonymous and non-synonymous changes in gp120 and gp41 (Supplementary Figure 1). The 7wpi_Luc clone was isolated before autologous neutralizing antibodies were detected at 14 wpi, and is likely to be a close approximation of the T/F virus (15). The temporal characterization shows that an accumulation of non-synonymous mutations is readily noticeable just after the 1st year of infection in the 54 wpi envs and continues to change across the entire gp160 in subsequent envs.

### CAP257 Env Immunogen Selection

Given the unique temporal neutralization breadth Waves in CAP257 targeting distinct epitopes, our initial goal for rabbit immunogenicity studies was to screen envelope clones from each Wave as vaccine candidates based primarily on neutralizing antibody titers. In particular, we wanted to determine if clones from one or more of the Waves would elicit higher antibody titers, broader neutralizing responses, or both when compared to other clones. In addition to the early env 7wpi_Luc, we selected envelopes from time points preceding the peak of each neutralization breadth Wave at 27, 30, 54, 93, and 174 wpi and used these to contruct gp140 trimers (Tables 1, 2, Supplementary Figure 1). At the time of this study, we constructed the gp140 trimeric Env proteins by eliminating the intersubunit proteolytic cleavage site (28–30), a commonly used approach before native-like Env designs were widely used, such as SOSIP mutations (31), or the stabilized cleavage-independent, native flexibly linked (NFL) trimers (32–34), or uncleaved prefusion-optimized trimers (35).

**Table 1:** Rabbit co-immunization strategies.

**Rabbit group (*n* = 6)**	**Imm # 1 (Week 0)**		**Imm # 2 (Week 4)**		**Imm # 3 (Week 12)**		**Imm # 4 (Week 20)**	
	**DNA**	**Protein**	**DNA**	**Protein**	**DNA**	**Protein**	**DNA**	**Protein**
7–30 wpi	7wpi_ Luc	7wpi_ Luc	7wpi_ Luc	7wpi_ Luc	30wpi_0_S4T5	30wpi_0_S4T5	30wpi_0_S4T5	30wpi_0_S4T5
	27wpi_3BI		27wpi_3BI		30wpi_S1T4		30wpi_S1T4	
	27wpi_6B		27wpi_6B		30wpi_S5T6		30wpi_S5T6	
54 wpi	54wpi_D	54wpi_D	54wpi_D	54wpi_D	54wpi_D	54wpi_D	54wpi_D	54wpi_D
					54wpi_C		54wpi_C	
					54wpi_G		54wpi_G	
93 wpi	93wpi_F12	93wpi_F12	93wpi_F12	93wpi_F12	93wpi_F12	93wpi_F12	93wpi_F12	93wpi_F12
					93wpi_E7		93wpi_E7	
174 wpi	174wpi_A9	174wpi_A9	174wpi_A9	174wpi_A9	174wpi_A9	174wpi_A9	174wpi_A9	174wpi_A9
	174wpi_B6		174wpi_B6		174wpi_G10		174wpi_G10	
					174wpi_H10		174wpi_H10	

**Table 2:** NHP co-immunization strategies.

**Group (*n* = 6)**	**Immunization timepoint**	**gp160 DNA**	**Trimeric gp140 protein**	**Time (weeks)**
No T/F prime	1, 2	54wpi_D	54wpi_D	0, 4
	3, 4	54wpi_D	54wpi_D	12, 20
		54wpi_C		
		54wpi_G		
	5, 6	93wpi_E7	93wpi_F12	32, 40
		93wpi_F12		
T/F prime	1, 2	7wpi_Luc	7wpi_Luc	0, 4
	3	54wpi_D	54wpi_D	12
	4	54wpi_D	54wpi_D	20
		54wpi_C		
		54wpi_G		
	5, 6	93wpi_E7	93wpi_F12	32, 40
		93wpi_F12		

**Figure 2:**
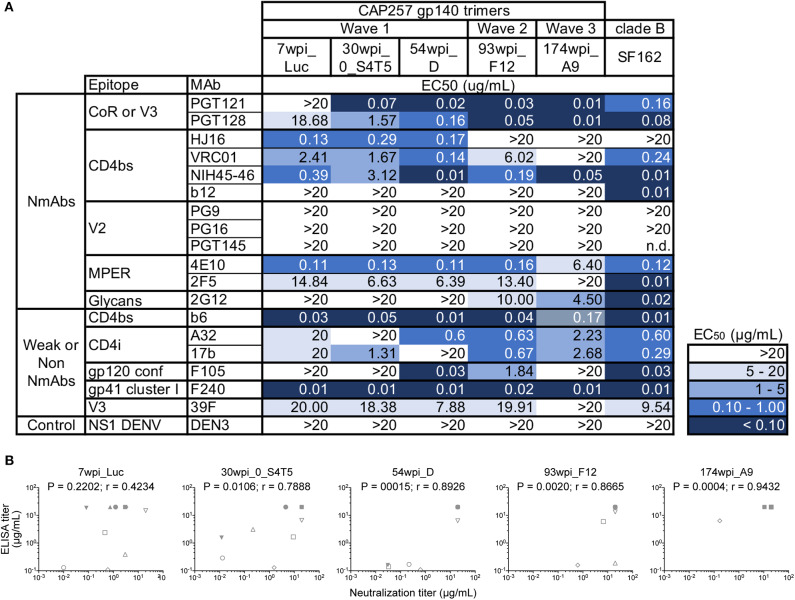
Midpoint ELISA binding responses of CAP257 gp140 trimers to nineteen monoclonal antibodies. **(A)** Trimeric gp140 proteins were captured onto ELISA plates and tested for binding of serially diluted antibodies (12 bNAbs and 6 weakly or non-neutralizing mAbs). The data are expressed as EC_50_ (μg/mL antibody concentration reaching 50% binding). **(B)** Correlations of ELISA binding responses of CAP257 gp140 trimers with neutralization by bNAbs (see Figure 3). *P* and *r*-values were determined by Spearman's correlation. The anti-Dengue mAb, DEN3, was used as a negative control on each assay plate.

**Figure 3:**
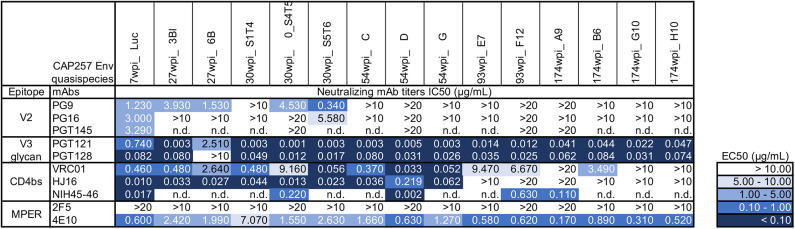
Antigenic characterization of gp160 DNA vaccines by neutralization of CAP257 Envs. All 15 gp160 envelope DNA vaccines were evaluated as pseudovirions (pSG3ΔEnv backbone) for neutralization sensitivity to 10 well-defined bNAbs in the TZM-bl assay format. The data are expressed as IC_50_ (μg/mL antibody concentration necessary to neutralize 50% of the infecting virus). The negative control is an anti-Dengue virus NS1 mAb (DEN3) which is tested in each neutralization experiment; DEN3 IC_50_ was consistently >20 μg/ml (not shown).

To antigenically characterize each of the purified uncleaved recombinant gp140 trimeric Env proteins representing CAP257 envelopes, several monoclonal antibodies directed to different regions, and epitopes were used to assess binding by ELISA (Figure 2). We also performed SPR to qualitatively compare neutralizing and non-neutralizing mAbs binding to CAP257 trimeric Envs. (Supplementary Figure 2). In both assay methods, binding was seen by N332-dependent bNAbs PGT121 and PGT128 (36) to all CAP257 gp140 trimers except 7wpi_Luc. Binding of the 54_D wpi CAP257 trimer was also seen in SPR by HJ16, VRC01, and NIH45-46 G54W, bNAbs that target the CD4 binding site (CD4bs), a conformational epitope on the HIV envelope. Differences in binding by CD4bs bNAbs were noticeable with NIH45-46 binding to all Env trimers, but little to no binding by VRC01 and HJ16 to Wave 2 and 3 trimers. The CD4bs bNAb b12 is not broadly reactive with clade C Envelopes, and no binding was seen by SPR (not shown) and IC_50_s were >20 in ELISA. No binding was seen by ELISA with V2 apex targeting PG9, PG16, or PGT145. The PG9 and PG16 epitopes are preferentially expressed on native trimeric HIV Env and bind strongly to Env expressed on the surface of infected cells. PG9 and PG16 can also bind with high affinity to some cleavage-defective trimers because their epitopes are accessible on the gp120 subunit conformation in the context of the stabilized trimeric spike rather than requiring gp120 cross-linking (37). Although the uncleaved nature of our gp140 trimers resulted in no recognition in SPR or ELISA binding experiments by these bNAbs (Figure 2 and Supplementary Figure 2), we expect that these epitopes are presented correctly by host antigen presenting cells expressing the gp160 DNA component of the co-immunization regimens (Figure 3). MPER-targeting 4E10 showed strong binding by ELISA to Wave 1 and 2 trimers. In contrast, only weak binding of 2F5 was seen in ELISA as expected because like most clade C viruses, none of the vaccine sequences contains the canonical 2F5 ELDKWA epitope. Glycans on the outer domain of gp120 are recognized by the bNAb 2G12. Only weak binding was detected in ELISA by 2G12 due to a critical glycan at position 295 that is generally lacking in clade C Envs (38). Binding by non-neutralizing antibodies is characteristic of uncleaved trimer structure, and binding was seen by both SPR and ELISA by the somatic variant of bNAb b12, non-neutralizing b6 against the 54wpi_D trimer. Binding was also seen by other weak or non-neutralizing mAbs of one or more CAP257 trimers, including F105 targeting a conformational epitope on gp120 and F240 indicating that cluster I gp41 epitopes are exposed on the CAP257 trimers.

The gp160 DNA vaccines were characterized by reconstruction as pseudoviruses bearing the selected native trimeric envelopes and assessed for neutralization sensitivity to ten bNAbs targeting known sites of Env vulnerability. The 7wpi_Luc env-bearing pseudovirions were weakly neutralized by all three V2 apex bNAbs tested. Of those, PG9 neutralized all but one of the early Wave 1 vaccines (30wpi_S1T4) (Figure 3), leaving all other Envs resistant to all V2 apex bNAbs. Also, bNAb neutralization of 30wpi_0_S4T5, 54wpi_D, 93wpi_F12, and 174wpi_A9 Env vaccines correlated with bNAb binding by ELISA, despite the absence of detectable binding in ELISA by V2 bNAbs (Figure 2B), All envelopes tested except 27wpi_6B, were very sensitive to V3 glycan bNAbs PGT121 and PGT128.

In summary, four vulnerable sites of the HIV-1 Env associated with bNAb sensitivity that we evaluated were represented by varying degrees on the majority of CAP257 trimers. The obvious exception is the V2 apex that was weakly neutralized by V2 bNAbs. Except for a single early Wave 1 trimer, consistent neutralization sensitivity is seen to the V3 glycan bNAbs. CD4bs epitopes on CAP257 Wave 1 and 2 trimers were sensitive to the bNAbs tested. The most consistent and strongest recognition across epitopes was seen by Envs appearing near peak breadth timepoints, specifically Wave 1, 54wpi and Wave 2, 93wpi trimers, and we have reported this observation earlier with clade B Envs (13, 14). Taken together, our antigenic characterization and the correlation between MAb binding to our trimers and neutralization of the envelope-bearing pseudoviruses suggest sufficient conformational integrity for utilization in vaccine strategies in animal models. Specifically, the co-immunizations using both gp160 DNA and gp140 Protein (Env trimers) as vaccine components, combine to present characteristics of natively expressed Envs to immunized animals.

### Immunogenicity Studies in Rabbits

We hypothesized that Envs from different Waves would have different immunogenic properties and, thus induce different outcomes when used as immunogens. Accordingly, we designed vaccine strategies for four groups of rabbits (consisting of six animals in each group) that were immunized with DNA and protein envelope immunogens that spanned CAP257 envs circulating during each of the three Waves of neutralization breadth (15). Clones from CAP257 quasispecies phylogeny were down-selected based on *in silico* sequence comparisons and *in vitro* autologous and heterologous neutralization patterns [see Figure 2 and (15)]. Each group contained unique envs representative of sequences associated with neutralization breadth within each Wave (Table 1). Together, the four groups included numerous clones representing the evolution of the quasispecies developed in CAP257.

**Figure 4:**
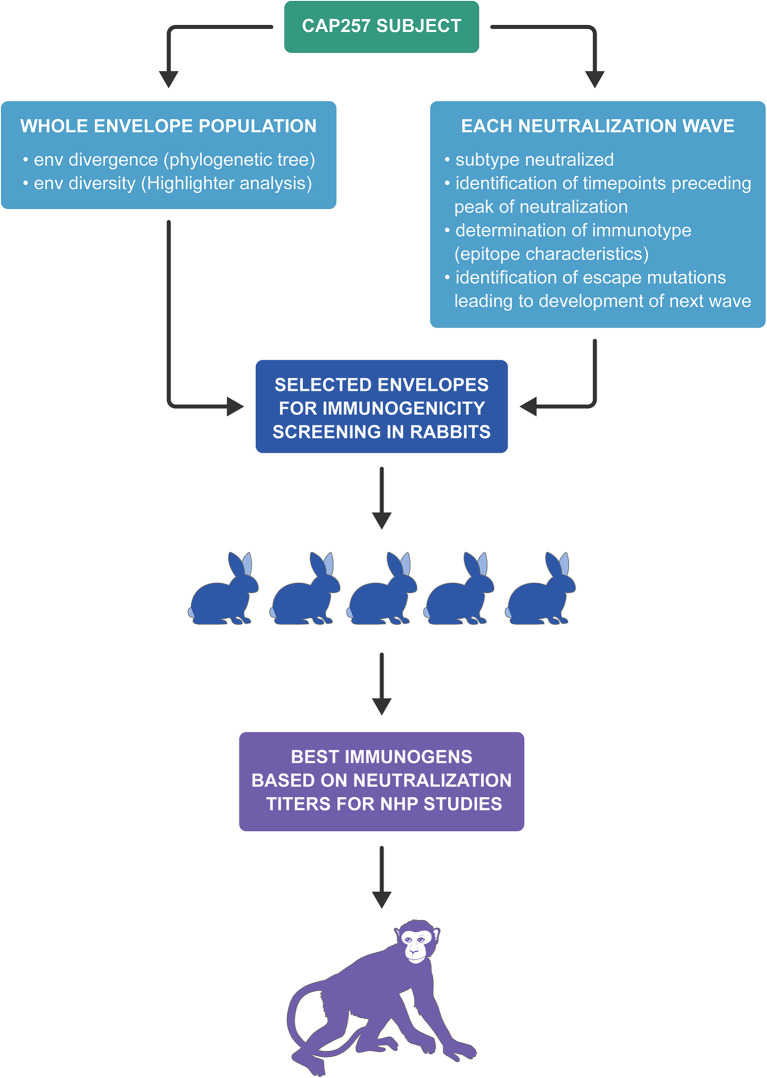
Flowchart depicting the overall scheme of CAP257 envelope selection for rabbit immunogenicity screening and NHP studies.

As previously described, an immunotype is an immunologically defined group of virus variants that share a signature motif (39) and our vaccine strategies are based on immunotypes. The first group of rabbits were immunized with envelopes from 7 to 30 wpi that were circulating early in Wave 1 when CAP257 NAbs were V1V2-directed, recognizing the N167 immunotype (with the exception of 30wpi_S1T4) and neutralization was dependent on N160 and K169 (15). The second group of rabbits were immunized with 54 wpi envs from Wave 1 that include escape variants (N167D mutation) within the V1V2 region that were associated with increased breadth in CAP257 (15). The third vaccine group was immunized with 93 wpi envelopes from the second Wave of neutralization in CAP257, which include an N279 immunotype and the presence of N276. The fourth vaccine group was immunized with 174 wpi envelopes from the third Wave of neutralization breadth even though Wave 3 envs include CD4bs NAb escapes due to the R456W mutation and Wave 2 escape variant envs due to the mutation N279D and the loss of N276 (15). A flowchart depicting the overall scheme of CAP257 envelope selection for immunogenicity studies is shown in Figure 4.

### Binding Antibody Responses in Rabbits

**Figure 5:**
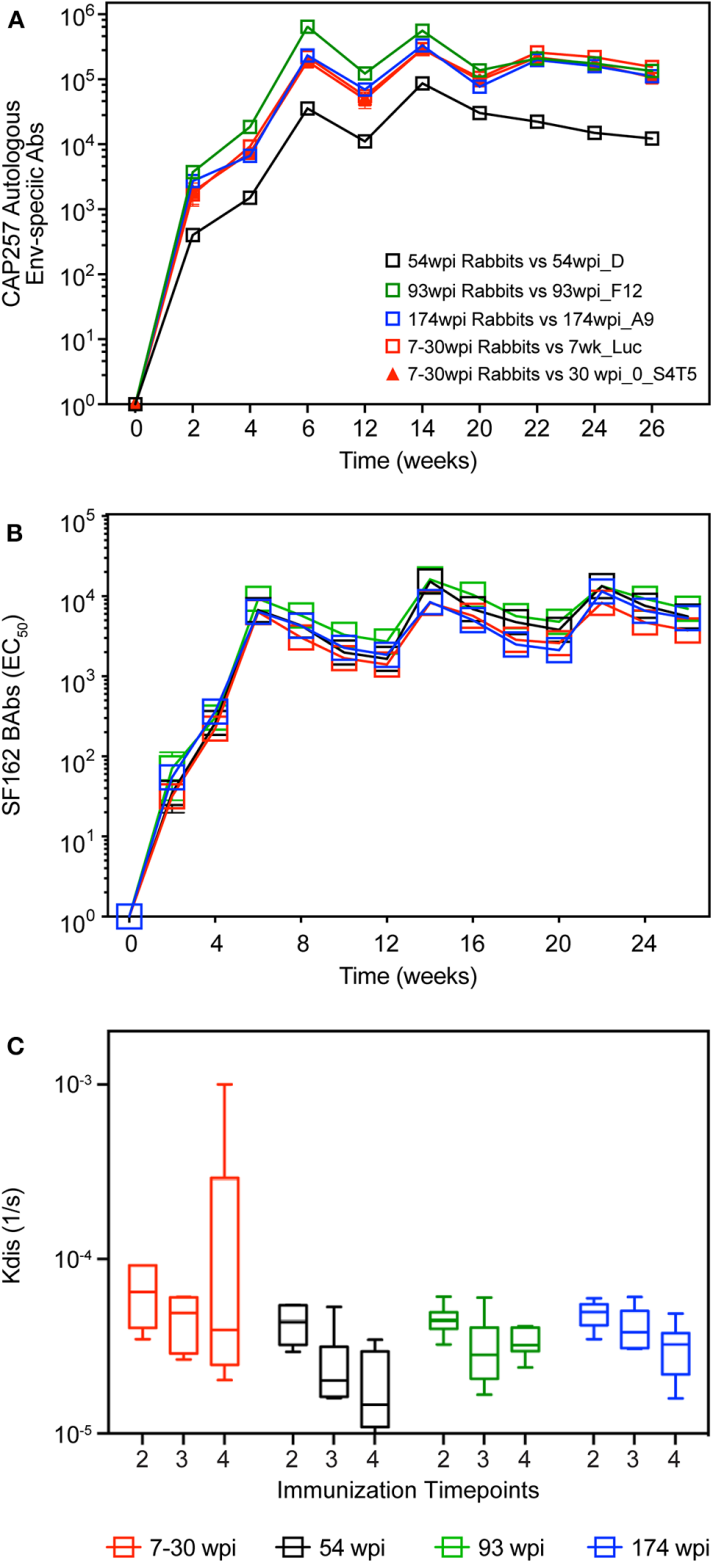
Longitudinal binding antibodies in rabbits. Serum samples from CAP257-vaccinated rabbits were tested longitudinally by midpoint ELISA for binding antibodies to **(A)** autologous env gp140 present in each vaccine and **(B)** HIV SF162 gp140. **(C)** Antibody affinity to HIV SF162 gp140 trimeric protein was measured by SPR. Data are *K*_dis_ values (1/s) after the second, third, and fourth co-immunizations (week 6, 14, and 22, respectively). Pre-immune sera was tested individually for each rabbit. Subsequently, a pooled pre-bleed was tested as a negative control in each assay. Statistical analyses of differences between the *k*_dis_ group responses were performed and were not found significant.

Using the four strategies described above and shown in Table 1, rabbits were co-immunized with gp160 envelope DNA and gp140 trimeric Env protein, in the presence of Rabbit GM-CSF (40) as DNA adjuvant and Adjuplex as protein adjuvant, respectively. Binding antibody responses (BAb) in each group were monitored longitudinally against soluble gp140 proteins from the autologous envs present in the vaccines (Figure 5A). Regardless of the antigen tested, midpoint Env-specific binding antibody titers were detected in all groups after only one DNA+Protein co-immunization (week 2), peaked with two vaccinations (week 6), and maintained similar titers with immunizations three and four (weeks 14 and 22). Surprisingly, the autologous binding titers against the 54wpi_D protein were lower compared to the other groups, but we could not rule out that this was due to technical issues with using in ELISA a different batch of purified protein from that used as the immunogen in this group of vaccinated rabbits. A consistent level of midpoint titers was measured longitudinally against the heterologous HIV-1 SF162 Env gp140 (Figure 5B), demonstrating comparable immunogenicity of the gp140 env immunogens in all groups.

Antibody affinity is a major characteristic of antibody-based vaccines and, since the evolution of the off-rates (*K*_dis_) is indicative of the antibody maturation as measured by a decrease in *K*_dis_ over time (28), we assessed the binding of SF162 gp140 trimeric protein to serum IgG in each vaccine group after the second, third and fourth co-immunizations (week 6, 14, and 22, respectively) using SPR. The *K*_dis_ values were not different between vaccine groups (Figure 5C), and due to higher than expected inter-animal variation within groups we did not detect a significant increase in antibody affinity over time.

### Epitope Targeting of Rabbit Antibodies

**Figure 6:**
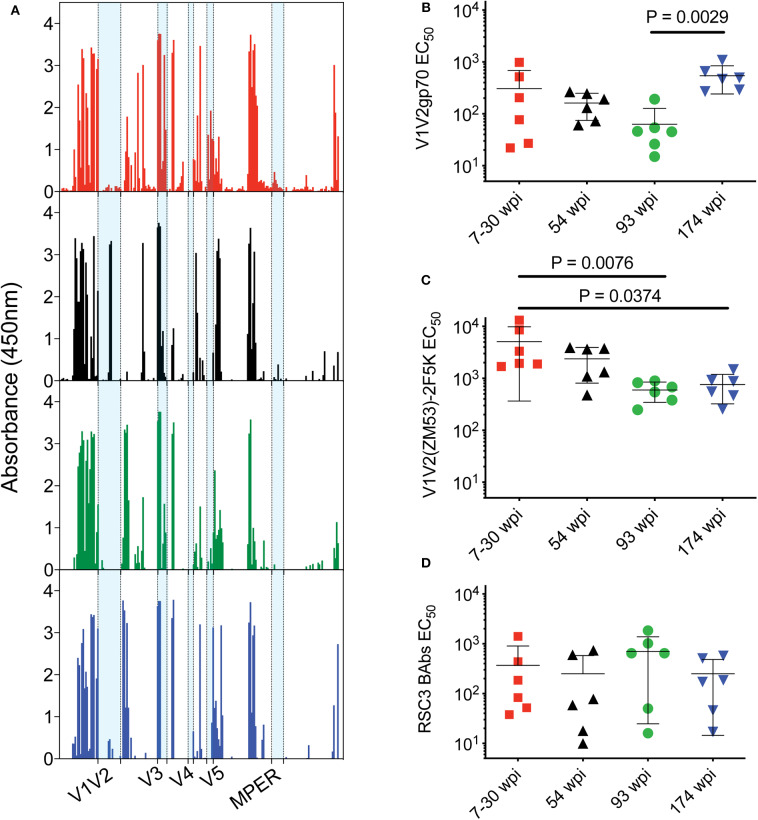
Epitope targeting of rabbit antibodies after the fourth immunization. **(A)** Pooled rabbit sera from each group were used to assess responses to linear epitopes contained in the clade C consensus Env peptides (15-mer with 11 aa overlap). Data are presented as absorbance (OD at 450 nm) after subtraction of background from naïve serum. V1V2 binding antibody responses were evaluated with **(B)** clade B gp70 V1V2 **(C)** V1V2(ZM53)-2F5K recombinant scaffold proteins. **(D)** CD4bs-specific binding antibody responses were evaluated with resurfaced gp120 recombinant core protein RSC3. Data in panels **(B–D)** are presented as mid-point titers. *P*-values determined by One-Way ANOVA with Kruskal–Wallis test for multiple comparisons. Lines indicate mean with SEM. Color coding for rabbit groups in panel **(A)** match groups indicated in panels **(B–D)**.

The targeting of the Env-specific binding Abs elicited in rabbits was first investigated with a gp160 scanning peptide ELISA (Figure 6A). Responses to linear epitopes contained in the clade C consensus Env overlapping peptides were determined in pooled rabbit sera from each vaccine group after the fourth immunization at week 22. Targeting of the C2 and 3′ region of C5 and gp41 was highest in the 7–30 wpi vaccine group. V1V2 peptide recognition was highest in the 54 wpi vaccine group, targeting two peptides located toward the 5′ end of the V2 region and containing the sequence ITTELRDKKQK (most probably the ITTE residues due to the overlap of 11 amino acids). Other regions of gp160 appear to be targeted similarly by all four vaccine strategies.

Anti-V1V2 binding antibodies were directly assessed using two different reagents by ELISA. The envs used in the 174 wpi vaccine strategy elicited higher V1V2- directed binding Abs compared to the 93 wpi strategy when assayed with a gp70 V1V2 recombinant protein by ELISA (*P* = 0.0029, Figure 6B). However, V1V2 loops do not fully retain their antigenicity in a gp70 scaffold context (41). When probed with a recombinant V1V2-scaffold protein [V1V2(ZM53)-2F5K] that presents conformational and linear epitopes, including the PG9 epitope (42), resulting titers were generally increased in all groups and the mean titer of rabbits in the 7–30 wpi vaccine strategy was greater than both the 93 wpi and the 174 wpi titers (*P* = 0.0076 and 0.0374, respectively, Figure 6C). Assessment of V1V2-directed antibody responses with these two different reagents suggests that both linear and conformational V1V2 antibodies were elicited by the vaccines. Moreover, higher binding titers elicited by the 7–30 wpi and 54 wpi envs against the V1V2-scaffold protein compared to the titers against the RSC3 gp120 core protein suggests the immunogenicity of the Wave 1 env immunogens used here may be representative of the Wave 1 viruses targeting V2 epitopes in CAP257. Although not significantly greater in a group-wise comparison, it is interesting to note that four of six rabbits immunized with the 93 wpi envs had higher binding titers to RSC3 than the majority of all other rabbits. Thus, this trend implies that 93 wpi vaccines used here and based on Wave 2 envelopes may be representative of the Wave 2 viruses leading to the CD4bs-directed neutralization breadth in the CAP257 donor (Figure 6D).

### Neutralizing Antibody Responses in Rabbits

**Figure 7:**
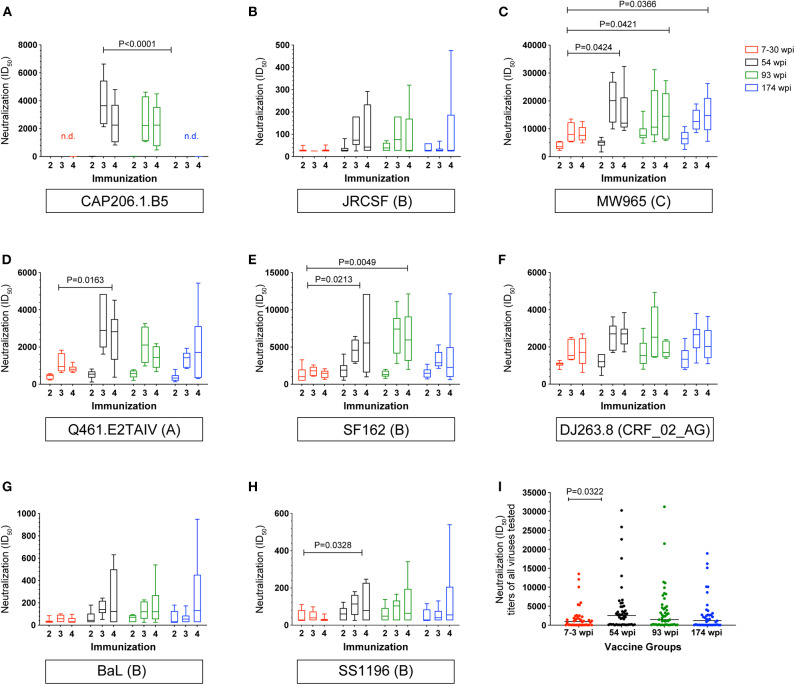
Longitudinal heterologous neutralizing antibodies elicited by CAP257 vaccine strategies in rabbits. **(A–H)** Rabbit serum samples after the second (week 6), third (week 14), and fourth immunization (week 22) were tested for neutralization of a panel of recombinant heterologous viruses in the TZM-bl assay. **(I)** Summary by group of all viruses neutralized. A decrease in RLU from a serum dilution <50 was considered as non-specific cell death and no neutralization. Data are expressed as ID_50_, serum dilution that neutralized 50% of the infecting virus. Box and whisker plots show mean ID_50_ for each group at each immunization timepoint shown with IQR and were calculated in GraphPad Prism 8.0; ANOVA, Kruskil–Wallis test; *P*-values are shown.

Neutralization titers in serum samples collected after the second, third, and fourth immunizations were measured against a panel of clade A, B, and C heterologous viruses (Figure 7 and Supplementary Figure 3A). The CAP257 env immunogens induced neutralizing antibodies against Tier 2 viruses CAP206.1.B5 (clade C) and JRCSF (clade B) (Figures 7A,B). Median heterologous neutralizing antibody (HNAb) titers against CAP206.1.B5 exceeding 1:2,000 in vaccinated rabbits were measured after three immunizations in the 54 wpi and the 93 wpi groups with the highest HNAbs induced by 54 wpi env immunogens (Figure 7A). Clade B, JRCSF HNAbs were primarily induced by the 54 and 93 wpi vaccines with minimal neutralization by only two rabbits in the 7–30 wpi group, and a single rabbit is accountable for the upper quartile and maximum data point shown in the 174 wpi group box and whisker plot (Figure 7B). Neutralization of Tier 1A viruses (MW965, Q461.E2^*^, and SF162) was very potent after only two immunizations (week 6 timepoint), and the highest HNAb titers were primarily seen in the 54 wpi vaccinated rabbits (Figures 7C–E). Tier 1B viruses, DJ263.8, BaL, and SS1196, that have more neutralization resistant phenotypes than Tier 1A viruses were also neutralized by rabbit sera over the course of the immunizations (Figures 7F–H). Overall, these analyses reveal that the 54 wpi and the 93 wpi vaccine strategies induced higher titers of NAbs compared to the 7–30 wpi or 174 wpi strategies against the majority of viruses tested. HNAb titers were boosted by the third immunization, but no further improvement was seen with the fourth immunization against either Tier 1 and 2 viruses. When compared, the median NAb titers after three immunizations vary significantly among the groups (*P* = 0.0227), and the 54 wpi env immunogens induced significantly higher NAb titers compared to 7–30 wpi envs (*P* = 0.0322, Figure 7I). Other viruses tested included Tier 1B virus ZM109 and Tier 2 viruses RHPA, CAP45, CAP61, and Du156 which were not neutralized (data not shown).

A complete longitudinal assessment of neutralization of SF162 was performed in the serum samples from each rabbit in order to provide an indication of the overall vaccine immunogenicity and its evolution following boosting (43) (Supplementary Figure 3B). Higher neutralizing titers of clade B SF162 were induced by the 54 wpi and the 93 wpi vaccine strategies compared to the 7–30 wpi strategy (*P* = 0.0049 and 0.0213, respectively).

Taken together, the immunogenicity studies in rabbits revealed that env immunogens chosen from CAP257 phylogeny replicated the bias of epitope targeting observed in individual CAP257, and that envs from Waves 1 and 2 induced Tier 2 HNAbs with the highest titers and greatest breadth of Tier 1 viruses. Therefore, based primarily on the neutralizing antibody titers from the rabbit experiments, we selected to test the CAP257 Env clones from 54 and 93 wpi in comparative immunogenicity groups of rhesus macaques.

### Immunogenicity Studies in Macaques

We immunized 12 adult rhesus macaques in two groups of six using the same co-immunization approach used in the rabbit groups described above. Although the 54 and 93 wpi Envs induced the highest titers of NAbs in separate groups of rabbits, based on *in vitro* antigenic characterization, the early 7 wpi clone was sensitive to V2 apex mAbs, and thus had the greatest potential of presenting the V2 apex epitopes when expressed endogenously in macaques with a DNA vaccine component. Therefore, we hypothesized that the T/F-like CAP257 Env isolated 7 wpi, if co-immunized as both DNA and protein as a priming step, would potentially “jump start” NAb induction before subsequent vaccination with 54 and 93 wpi CAP257 Envs.

To test our hypothesis, we established two vaccine strategies using co-immunization with DNA and gp140 (Table 2). The first of these was designated as the No T/F Prime group with macaques receiving one or more 54 wpi clones alone for four immunizations followed by two immunizations with 93 wpi Envs; the second was designated as T/F Prime group with macaques receiving two priming immunizations with the T/F-like 7 wpi Env followed by two immunizations with 54 wpi Envs then two immunizations with 93 wpi Envs. Macaques were immunized at weeks 0, 4, 12, 20, 32, and 40. At each immunization, macaques received 36 μg of plasmid gp160 DNA expressing one or more native env sequences via gene gun epidermal delivery simultaneously with an intramuscular delivery of 50 μg of at least one soluble gp140 trimeric protein formulated in Adjuplex adjuvant from the specified timepoint. We collected blood samples at regular intervals for monitoring antibody binding and neutralizing titers, epitope targeting, CD4 T helper follicular (T_FH_) activation, and antibody-dependent cellular cytotoxicity (ADCC).

### Binding Antibodies in Macaques

**Figure 8:**
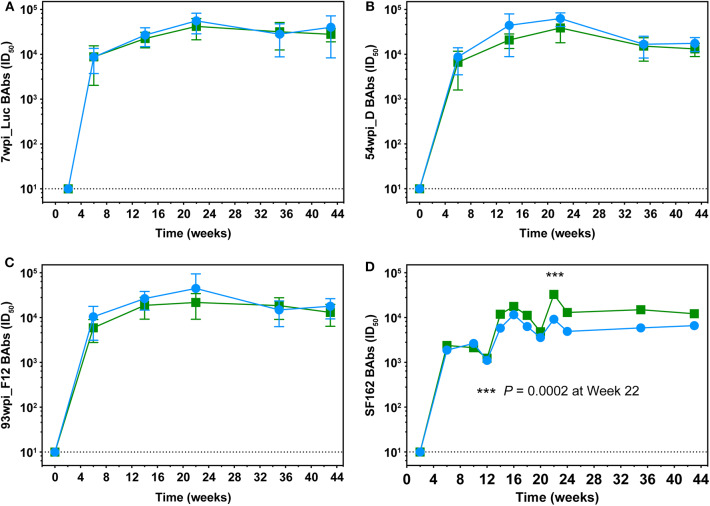
Longitudinal assessment of Env-specific binding antibodies in macaque sera. Longitudinal binding antibodies to autologous 7wpi_Luc **(A)**, 54wpi_D **(B)**, 93wpi_F12 **(C)**, gp140 trimeric proteins and to heterologous SF162 gp140 trimer **(D)** were determined by ELISA. Data are presented as mid-point titers. *P*-value in panel **(D)** was determined by RM 2-way ANOVA.

BAb responses in macaque sera were monitored longitudinally against the autologous gp140 immunogens. Regardless of the vaccine strategy, BAb titers specific for 7wpi_Luc Env and 54wpi_D Env were nearly identical in kinetics and magnitude reflecting the high degree of homology among the cloned Envs isolated during the 1st year of infection in CAP257 (Figures 8A,B). The 93wpi_F12 gp140 protein was given to each group only at immunizations 5 and 6, but BAbs were in the range of titers against the earlier Envs 7wpi_Luc and 54wpi_D (Figure 8C).

Midpoint heterologous clade B SF162 Env-specific binding antibody titers on the order of 10^3^ were detected in all animals in both groups after two DNA+Protein co-immunization and increased to ~10^4^ after the third immunization (Figure 8D). The only significant difference between the groups is noted at week 22 after the fourth immunization where the No T/F Prime group had higher BAb titers compared to the T/F Prime group (*P* = 0.0002, RM 2-way ANOVA). Overall, BAb titers did not increase in either group with the fifth and sixth immunizations with 93 wpi envelopes and remained at the approximate titer reached after the third vaccination.

An important characteristic of a protective vaccine is the establishment of long-lived plasma B cells. The durability of antigen recognition from these Env-based immunogens was seen when a single macaque (24,383) was available for a blood draw 2 years after immunizations had ceased in this study. Plasma BAb titers at that time for 24,383 were >10^4^ in ELISA measured against heterologous SF162 and against autologous Envs 54wpi_D and 93wpi_F12 (Supplementary Figure 4).

### Epitope Targeting of Plasma Antibodies in Macaques

**Figure 9:**
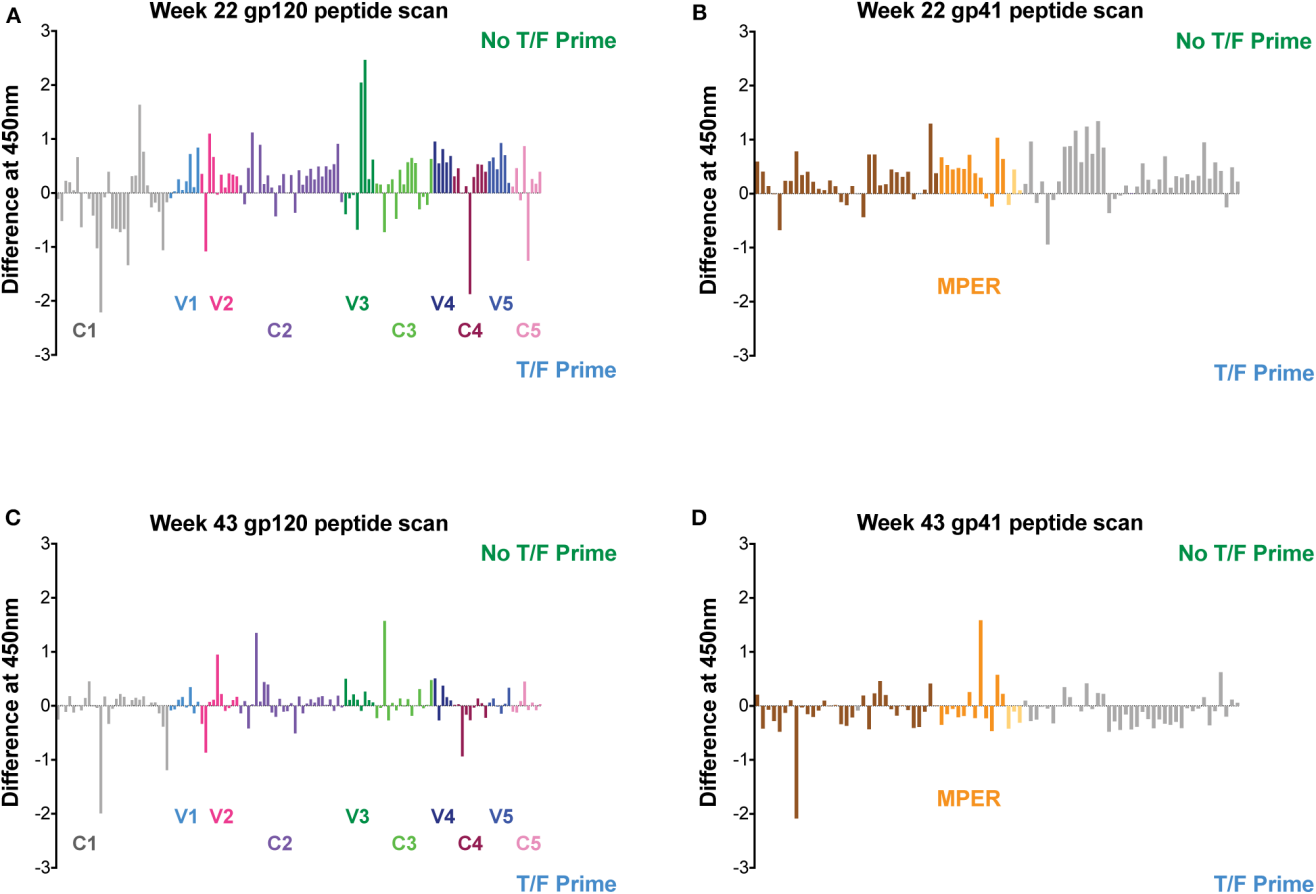
Linear epitope targeting of plasma antibodies in macaques. Pooled macaque plasma was used in ELISA for binding against clade C consensus Env overlapping peptides after the fourth (at week 22) and sixth (at week 43) immunizations. Data plotted is the difference between each vaccine group and shown as the magnitude of signal against each peptide. Responses against regions of gp120 are shown in panels **(A,C)**. Panels **(B,D)** show responses against regions of gp41.

The targeting of the Env-specific BAbs elicited in macaques was evaluated with a gp160 scanning peptide ELISA. Responses to linear epitopes contained in the clade C consensus Env overlapping peptides were determined in pooled macaque sera from each vaccine group after the fourth and sixth immunizations at weeks 22 and 43, respectively. The differences between the vaccine groups for binding to each peptide were calculated and plotted as shown in Figure 9. Although similar levels of autologous and heterologous binding Abs against gp140 Env protein immunogens were measured in both vaccine groups (Figures 8A–D), the results of the peptide scan suggest vaccine-specific regional targeting and differences in overall magnitude in the polyclonal response to Env. Excluding the T/F-like 7wpi_Luc immunogen in the No T/F Prime vaccine strategy resulted in a greater magnitude of gp120 and gp41 peptide responses than corresponding responses measured in the T/F Prime group after four immunizations (compare Figures 9A,B to C,D, *P* < 0.0001). Higher and more numerous peptide responses were induced in No T/F Prime group macaques in both in the variable regions V1V2, V3, V4, and V5 and in the constant regions C2, C3, and C5 of gp120. In contrast, the T/F Prime vaccine group had stronger gp120 responses in the constant regions C1 and C4. The magnitude of the responses was not improved in either strategy after the additional fifth and sixth immunizations, and instead gp120 responses (Figures 9A–C, *P* = 0.0161) and gp41 responses (Figures 9B–D, *P* < 0.0001) were significantly reduced. These data combined with BAbs data above suggest that neither including the T/F Env immunogen or giving more than four immunizations improved antibody responses to linear Env epitopes. Although V1V2 region linear epitope responses were greater and more numerous in the No T/F Prime group compared to the T/F Prime group, when tested for binding to the V1V2-scaffold protein that presents both conformational and linear epitopes, there was no difference between the vaccine groups (Supplementary Figure 5). Further characterization of macaque sera was performed in ELISA with the resurfaced core gp120 (RSC3) protein (44) to assess the fraction of antibody responses that targeted the CD4 binding site (CD4bs). A range of midpoint titers among vaccinated macaques was measured with no difference between groups in CD4bs targeting (Supplementary Figure 5).

### Neutralizing Antibodies in Macaques

**Figure 10:**
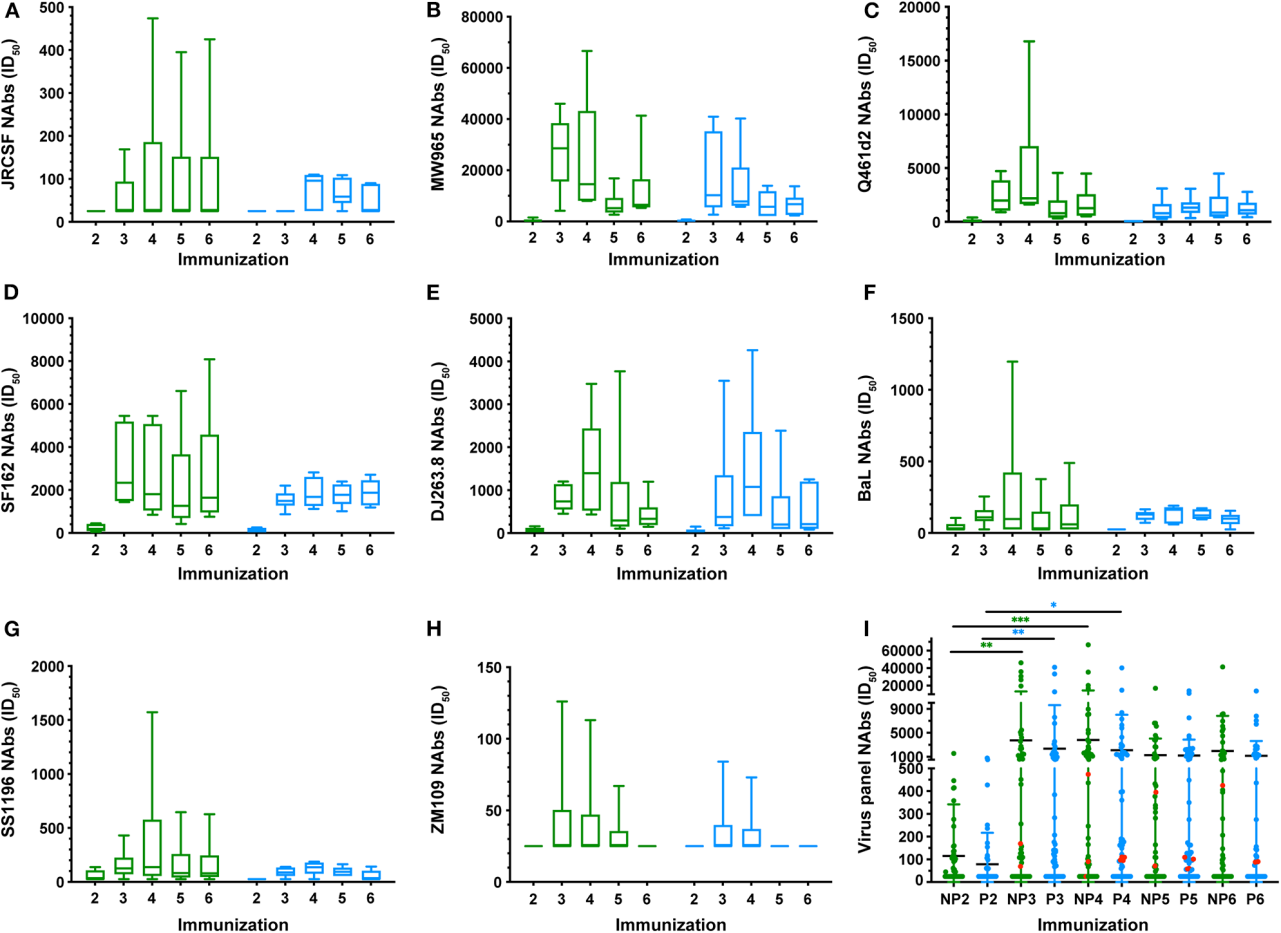
Longitudinal heterologous neutralizing antibodies elicited by CAP257 vaccine strategies in macaques. **(A–H)** Serum samples after the second (week 6), third (week 14), fourth (week 22), fifth (week 35), and sixth (week 43) immunization were tested for neutralization of a panel of recombinant heterologous viruses in the TZM-bl assay. **(I)** Summary by group and immunization number of all viruses neutralized. Lines are mean and SD. A decrease in RLU from a serum dilution <50 was considered as non-specific cell death and no neutralization. Data are expressed as ID_50_, serum dilution that neutralized 50% of the infecting virus. Box and whisker plots show mean ID50 for each group at each immunization timepoint shown with IQR and were calculated in GraphPad Prism 8.0; ANOVA, Kruskil-Wallis test; *P*-values are shown.

A panel of pseudoviruses representative of envelopes from Tiers 1A, 1B, and 2, clades A, B, and C were tested for heterologous neutralization after the second, third, fourth, fifth, and sixth immunizations (Figure 10, Supplementary Figure 6). Mean titers of each group over the course of the immunizations for neutralization of all viruses were comparable. However, the No T/F Prime group induced statistically higher NAb titers against clade A, Tier 1A Q461.E2^*^ after the fourth immunization (Figure 10C, *P* = 0.0123, RM 2-way ANOVA) and against clade B, Tier 1A SF162 after the third immunization (Figure 10D, *P* = 0.0285, RM 2-way ANOVA) compared to the T/F Prime group. Also, comparing the No T/F Prime and T/F Prime groups for neutralization of each virus across all six immunizations, Tier 1A, clade C, MW965, and CRF_02_AG, DJ263.8 were neutralized more potently by sera from No T/F Prime macaques (Figures 10B,E, *P* = 0.0004 and 0.0021, respectively, 2-way ANOVA, Multiple Comparisons). All other viruses were neutralized with comparable potency by both vaccine groups. When comparing overall NAb titers in each group at each immunization timepoint, the greatest improvement occurred with the second and third immunizations in both groups, and additional immunizations and the introduction of the 93 wpi vaccines (immunizations 5 and 6) did not improve neutralization potency or breadth (Figure 10I). NAb titers against Tier 2 JRCSF were detected in three animals from each group, but this activity appeared earlier (post-immunization 3 vs. 4) and was greater in the No T/F Prime group (see red data points in Figure 10I). No autologous NAbs were elicited against either the T/F-like 7wpi_Luc or 54wpi_D Env (data not shown).

### Macaque T_FH_ Responses

**Figure 11:**
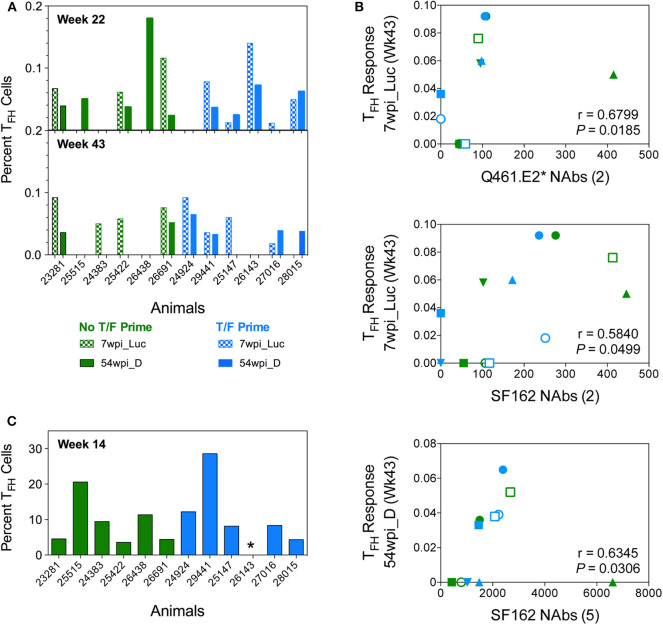
Env-specific T_FH_ cells are quantified in LN of vaccinated NHPs. The percentage of functional CD4+ T_FH_ cells stimulated by vaccine immunogens in inguinal lymph nodes of vaccinated macaques was assessed. **(A)** Lymphocytes collected 2 weeks after 4 (week 22) and 6 (week 43) immunizations were stimulated with both 7wpi_Luc and 54wpi_D Env proteins and analyzed by conventional intracellular staining where T_FH_ cells were defined as CD3+CD4+CD95+, PD-1hi, and Env-specific responses were measured by IL-21 and IFNg. **(B)** Correlations of immunogen-specific T_FH_ responses with neutralization activity in sera of vaccinated macaques. The number of immunizations given are shown in parenthesis in the *x*-axis title. *P* and *r* values were determined by Spearman's correlation. **(C)** Lymphocytes collected 2 weeks after 3 (week 14) immunizations were assessed after stimulation with 54 wpi_D Env protein. T_FH_ cell subpopulations were defined as CD20–CD3+CD4+, PD-1hi, CXCR5hi. Data shown is the percentage of functional CD4+ T_FH_ cells identified as the OX40+ and CD25+ subset. *Indicated no data for this NHP. No T/F Prime group in green and T/F Prime group in blue for all panels.

Envelope-specific T_FH_ responses were assessed in draining lymph nodes after the third, fourth, and sixth immunizations (Figure 11A). An initial analysis of T_FH_ responses was performed after the fourth and sixth vaccinations by quantifying either CXCR5^+^ or ICOS^+^, PD-1^hi^ populations using conventional intracellular flow cytometry staining (14). Using this analysis method, regardless of the immunization timepoint or the trimeric Env immunogen tested, the T_FH_ responses elicited by the two vaccine strategies were not statistically different from each other (*P* > 0.05, Mann–Whitney test). However, immunogen-specific T_FH_ responses for 7wpi_Luc measured after the last vaccination at week 43 were positively correlated with neutralization of Q461.E2^*^ (*P* = 0.0185, *r* = 0.6799) and of SF162 (*P* = 0.0499, *r* = 0.5840) after the second immunization (Figure 11B). In addition, immunogen-specific T_FH_ reponses for 54wpi_D measured at week 43 positively correlated with SF162 after the fifth immunization (*P* = 0.0306, *r* = 0.6345).

Subsequently, we used a different approach identifying T_FH_ cells as PD-1^hi^, CXCR5^hi^ and assessing highly upregulated activation-induced markers (AIM) on the surface of GC T_FH_ cells after immunogen stimulation (45). Unfortunately, due to limited samples, we were only able to probe T_FH_ responses specific for the 54wpi_D env immunogen after the third immunization using this method (Figure 11C). The T_FH_ responses elicited by the two vaccine strategies were not statistically different from each other (*P* > 0.05, Mann–Whitney test).

### Macaque Serum ADCC Activity

**Figure 12:**
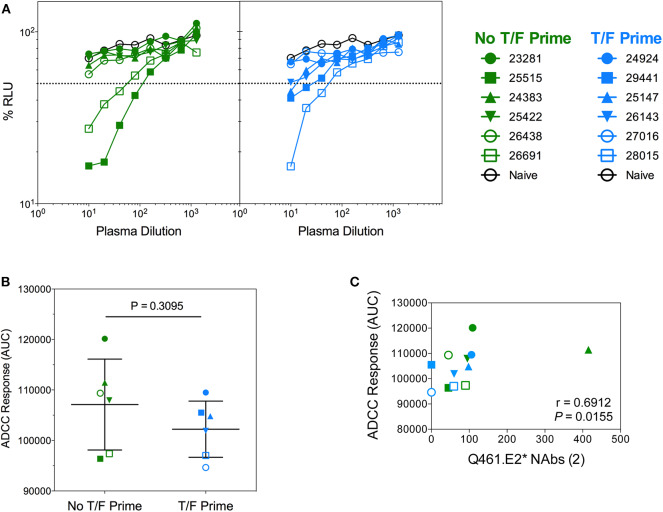
Antibody-dependent cellular cytoxicity (ADCC) of NHP sera against Tier 2 SHIV_SF162P3_ infected cells. **(A)** Serially diluted plasma samples were tested for ADCC activity in a SHIV_SF162P3_ infected cell assay. ADCC responses were measured as the dose-dependent loss of luciferase activity in relative light units (RLU) after incubation in comparison to control wells containing NK cells and either infected (maximal) or uninfected (background) CEM.NKR-CCR5-sLTR-Luc cells in the absence of antibody. The dotted line indicates half-maximal lysis of infected cells. **(B)** %RLU values were used to determine area under the curve (AUC) values. *P*-value was determined by Mann–Whitney test. **(C)** Correlation of ADCC responses and neutralization of Q461.E2* virus. The number of immunizations is indicated in parentheses in the *x*-axis title. *P* and *r*-values were determined by Spearman's correlation.

Vaccine-induced functional antibody responses are becoming recognized as an important partner with neutralization in antibody protection *in vivo* (46–48). In vaccine pre-clinical and clinical trials and in natural infection, the importance of ADCC is well-documented (49). To evaluate ADCC activity in vaccinated macaques, we assayed plasma samples against Tier 2 SHIV_SF162P3_-infected cells from each group at week 22 (Figure 12A). ADCC activity in both groups was comparable (Figure 12B), and sera antibodies from three animals were able to mediate >50% ADCC activity (defined as the plasma dilution giving >50% reduction in RLU). These animals, (28,015, 25,515, and 26,691) also had some degree of T_FH_ activation (Figure 11) but had no neutralization of the autologous viruses. The macaque ADCC activity against Tier 2 SHIV_SF162P3_-infected cells correlated with neutralization of Q461.E2^*^ (*P* = 0.0155, *r* = 0.6912; Figure 12C) after the second immunization. Importantly, antibodies induced by CAP257 Env immunogens derived from clade C viruses killed cells infected with a heterologous, clade B Tier 2 SHIV_SF162P3_, although there was no neutralization against the virus (data not shown).

## Discussion

The design of HIV immunogens effective at addressing the low immunogenicity of conserved envelope neutralization determinants that are the targets of bNAbs remains a significant obstacle to an effective HIV vaccine. CAP257 developed three temporal Waves of neutralization breadth that targeted different epitopes (15) and were elicited by potentially distinct lineages. Using natural quasispecies envelope sequences that evolved during the development of breadth in CAP257, we performed comparative rabbit and macaque vaccine studies with selected diverging envelope clones in an effort to identify sources of immunogens capable of eliciting NAb breath in vaccinees. These experiments revealed that pre-peak timepoints during breadth development at ~1–2 years post-infection may be a promising source of clade C envelope immunogens similar to our findings with clade B envelopes in rabbits (13) and in rhesus macaques (14).

Our vaccine regimen is a co-immunization combining a gp160 DNA component and a gp140 trimeric protein. The full-length gp160 DNA delivers natively expressed protein *in vivo* while the gp140 Env protein provides additional priming of a T cell dependent type of B cell response to stimulate isotype switching and affinity maturation during antibody development. This approach optimizes immunogen presentation and elicits moderately broad neutralizing antibody titers in rabbits (13) and macaques, including Tier 2 autologous NAbs (14, 50). Moreover, it induces immunogen-specific T_FH_ responses in macaques (14) and ADCC activity (42). The DNA+Protein co-immunization strategy has also been used with SIV immunogens and, when associated with TLR4+7 adjuvants, was able to elicit humoral and cellular immune responses that contributed to control of viremia in a heterologous mucosal repeated challenge (51). The potential of the DNA+Protein co-immunization regimen was further enhanced by the recent results of a human clinical trial showing that Env protein co-immunization with DNA or NYVAC elicted faster, broader and more potent binding and Tier 1 neutralizing antibodies compared to a prime/boost approach (52, 53). Nonetheless, whether the same envelopes from the CAP257 subject would be even more immunogenic if constructed in a native-like platform for expression is unknown. At the time of development of BG505 SOSIP, the titers of autologous Tier 2 antibody responses were very comparable to titers induced with the uncleaved trimeric gp140s we were using to co-immunize macaques. Indeed, the neutralizing titers against Tier 1 and 2 autologous and heterologous viruses were higher. In the ensuing years, much progress has been made to improve the antigenicity and immunogenicity of native-like constrained Envs, and it is likely that we could improve the antibody responses using the CAP257 Envs expressed in a different platform. Attempts have been made by other groups to develop SOSIP trimers of CAP257 Envs without success (personal communication with P. Moore). However, efforts devoted to new constructs of clade C native envelopes, especially derived from the rich CAPRISA cohort of subjects with broad and potent breadth could provide the field with new immunogen candidates.

The tested vaccine strategies replicated several characteristics of the neutralization Waves from which the envelopes were isolated. Wave 1 vaccine envelopes targeted V1V2 epitopes and were sensitive to both V2-directed and CD4bs-directed bNAbs in agreement with Wibmer et al. (15). Rabbits immunized with 54wpi envelopes developed strong binding and neutralization responses. In the context of our study, vaccination with early envelope variants (7–30 wpi) from Wave 1 did not induce high or broad neutralizing responses in rabbits despite the potential for V2 apex antigenicity. These results are not completely unexpected since this early lineage mainly elicited strain-specific NAbs in CAP257 subject (15). We hypothesize that, since CAP257 developed three temporal Waves of neutralization breadth that targeted different epitopes (15) and were elicited by potentially distinct lineages, the induction of this early lineage was not sufficient to drive the development of neutralization breadth and induction of multiple lineages could be more efficient (17, 21). Indeed, priming with 7wpi_Luc in macaques did not effectively prime neutralization breadth. Wave 2 envelopes did not target V1V2 epitopes and were only sensitive to CD4bs-directed bNAbs in agreement with our previous findings (15). The 93 wpi Env vaccine strategy which contained immunogens from a timepoint ~7 months preceding the peak of Wave 2 neutralization breadth was also highly immunogenic in rabbits and elicited similar binding and neutralizing antibody titers to the 54wpi Env vaccine. The fourth vaccine group was immunized with 174 wpi envelopes from the third Wave of neutralization breadth even though Wave 3 envs include CD4bs NAb escapes due to the R456W mutation and Wave 2 escape variant envs due to the mutation N279D and the loss of N276. Wave 3 vaccine envelopes were largely resistant to V2-directed and CD4bs-directed bNAbs and were immunogenic but were not as potent as the 54 and 93 wpi Env vaccines.

Similar to the clade C MVA prime—gp120 protein boost vaccine study by Pollara et al. (54), we found that rabbits and macaques vaccinated with the same clade C envelope immunogens elicited equivalent levels of binding and neutralizing antibodies. However, the neutralization titers achieved in both animal models, despite high titers against Tier 1A viruses, did not replicate the neutralization breadth observed in the infected subject, the source of the immunogens (15). Several reasons, individually or in combination, could play a role in this discrepancy. The much greater antigenic stimulation associated with consistently high viral loads and exposure to a swarm of variants in the CAP257 cohort participant may be a major factor in this difference. In addition, the selected envelope vaccines are the dominant env clones from the quasispecies viral populations sampled at each timepoint whereas a minority variant revealed by deep sequencing drove the development of CD4bs neutralizing antibody responses in subject CAP257. This important point was discovered after the selection, design, and initiation of the experiments described here. It could also potentially explain why our vaccines did not elicit the neutralization breadth of CAP257, since all envelope vaccines from 7 to 93 wpi possess a potential N-linked glycosylation site at position 276, whereas the development of CD4bs bNAbs was likely driven by envelopes with a glycan hole at that position (18). The lack of autologous neutralization was surprising, but it is also likely that immunogens being derived from the dominant env clones without the glycan hole, may explain the absence of neutralizing activity. Although the env clones, when made as pseudoviruses were sensitive to potent monoclonal bNAbs, it is unlikely that polyclonal antibodies, even with some Tier 2 heterologous neutralization activity would neutralize the CAP257 quasispecies.

Based on our results from our *in vitro* antigenicity studies with mAbs, and because our vaccines elicited strong Tier 1A NAbs and moderate Tier 1B NAbs, we hypothesize that the gp140 trimeric protein vaccines are in open and/or intermediate conformational states (43) which do not favor the elicitation of Tier 2 NAbs. We propose that this adverse conformation may have been promoted by the Adjuplex adjuvant. Adjuplex is a carbomer-lecithin adjuvant that elicits balanced Th1/Th2 humoral responses as well as cellular responses in vaccinated mice (55) and was shown to also elicit B and T cell responses in vaccinated macaques (14). However, it was recently suggested that Adjuplex may skew responses toward the development of Tier 1 neutralizing antibodies by inducing conformation changes that imbalance antigenic quaternary structures (56). It was also just shown that Adjuplex did not elicit broad NAbs compared to adjuvant 3M-052 (57). Despite these limitations, the neutralization in the TZM-bl assay system of two Tier 1B and two Tier 2 viruses by vaccinees' sera is a promising step forward, since Tier 1B virus neutralizing titers track more with Tier 2 than with Tier 1A viruses (58).

ADCC activity was identified as a correlate of reduced risk of HIV infection in the RV144 clinical trial (59) and several preclinical vaccine studies have correlated ADCC responses with protection from SHIV or SIV challenge or with reduction of viremia (2, 60–63). However, it has also been recently shown that protection from autologous challenge could be achieved without the development of ADCC responses (64). In the current study, some macaques immunized with CAP257 envelope immunogens elicited ADCC responses. Here, these ADCC responses correlated with heterologous neutralization of Tier 1A, clade A virus Q461.E2^*^ and Tier 1B, clade B SS1196 viruses after the second immunization and of Tier 2, clade B JRCSF virus after the third immunization. Thus, it is encouraging that five NHPs developed 50% or greater ADCC activity and that it was positively associated with heterologous neutralization.

Development of high quality and long-lived humoral responses requires help from T_FH_ cells in germinal centers (GC) of lymph nodes. T_FH_ cells play there a critical role in the generation of a protective antibody response as they control events that drive B cell proliferation, affinity maturation and immunoglobin class-switching. In the SIV infection model, neutralization breadth correlated with GC T_FH_ responses (65). Furthermore, early T_FH_ responses measured 3 days post-priming, were recently associated with control of viremia in NHPs primed with Ad5 SIV vaccine (66). We showed previously that clade B Env DNA+Protein co-immunization elicited Env-specific T_FH_ responses in macaques and that these responses correlated with autologous Tier 2 neutralization titers (14). In the current study, clade C immunogens also elicited immunogen-specific T_FH_ responses that correlated with neutralization of hetelogous neutralization of Tier 1A clade A Q461.E2^*^ (after the second immunization) and clade B SF162 (after the second and fifth immunizations). Thus, the association between the elicited T_FH_ responses and heterologous neutralization is a positive feature of the DNA and protein co-immunization vaccination strategy. Further improvements could lead to increased T_FH_ responses, as several factors can affect the level and quality of T_FH_ responses including the nature of the priming agent (DNA only or with protein) (67) and the level of GC T-B cell interactions since GC B cell frequency after the last protein boost was the best predictor for development of potent Tier 2 autologous NAbs (45).

The distinct wave pattern of NAb breadth to multiple epitopes in CAP257 provided a unique source of clade C envelopes from which we were able to parse breadth timepoints and test for the best source of immunogens in multiple rabbit vaccine groups. In this clade C subject and similar to our previous findings with clade B envelopes (13), we found that breadth timepoints—~1–2 years post-infection—were the best immunogen source. Subsequently, in macaques, using two sequential timepoints from early breadth development in CAP257, co-immunizations of DNA and protein induced neutralizing antibodies, immunogen-specific T_FH_ activation and functional Fc-mediated ADCC responses against SHIV-infected target cells. Using a very early T/F envelope isolated 7 weeks post-infection as a prime did not increase breadth or potency of the elicited humoral and cellular immune responses. Using an envelope isolated more than 3 years post-infection in additional immunizations also did not enhance responses. These rabbit and macaque immunogenicity data demonstrate that our approach to down-select specific timepoints along the developmental path of neutralization breadth is not restricted to clade B envelopes and can be extended to other HIV clades. It is also noteworthy that in CAP257, the envelopes derived from the 1 year post-infection timepoint belong to the neutralization Wave that targeted the V2 region of Env, but the NHP polyclonal serum Ab responses here were fairly evenly divided between CD4bs and V1V2 region epitopes. We did not perform epitope mapping experiments to distinguish whether V2 or CD4bs targeting accounted for specific fractions of the NAb titers. Nonetheless, whether these modest responses can be improved with regimens that include Gag or adenovirus-vectored vaccines in order to protect against a heterologous Tier 2 SHIV challenge warrants investigation.

## Materials and Methods

### Ethics Statement

CAP257 is a participant enrolled in the CAPRISA 002 Acute Infection study, established in 2004 in Kwa-Zulu Natal, South Africa. The CAPRISA 002 Acute Infection study was reviewed and approved by the research ethics committees of the University of KwaZulu-Natal (E013/ 04), the University of Cape Town (025/2004), and the University of the Witwatersrand (MM040202). CAP257, an adult, provided written informed consent. CAP257 is a clade C, HIV-1 infected individual from the CAPRISA 002 Acute Infection Cohort, which is comprised of women at high risk of HIV-1 infection in Kwa-Zulu Natal, South Africa. Plasma samples were available over a period of 4.5 years post-infection (YPI), after which CAP257 started anti-retroviral therapy. During the time of observation [7 weeks post-infection (wpi) to 4.5 YPI], CAP257 had an average plasma viral load of 60,784 copies/mL and an average CD4 count of 498 cells/μL. She did not show signs of AIDS-defining illnesses.

The studies were carried out in accordance with the recommendations described in the Guide for the Care and Use of Laboratory Animals of the National Institutes of Health. All animal work was approved by the Oregon Health and Science University (OHSU) Institutional Animal Care and Use Committee (IACUC). Animal facilities at the Oregon National Primate Research Center (ONPRC) are accredited by the American Association for Accreditation of Laboratory Animal Care. All efforts were made to minimize animal suffering and all procedures involving potential pain were performed with the appropriate anesthetic or analgesic. The number of animals used in these studies was scientifically justified based on statistical analyses of immunological outcomes.

### Rabbit Immunizations

A total of 24 Female New Zealand White rabbits (Western Oregon Rabbit Company, Philomath, OR) were housed at the Oregon National Primate Research Center (ONPRC) in Beaverton, OR. Immunizations were performed on rabbits of 6 pounds of weight or greater. All procedures were performed according to rules and protocols approved by the Institutional Animal Care and Use Committee at OHSU.

Rabbits (six per group) were co-immunized with DNA and protein at weeks 0, 4, 12, and 20. A total of 36 μg DNA was delivered epidermally by Particle Mediated Epidermal Delivery (PMED gene gun) XR-1 device (PowderMed, Oxford, UK), at a pressure of 400 psi, in 18 immunizations of 2 μg DNA each given in clusters of three non-overlapping positions at six shaven sites (lower back, inside of back legs, and abdomen). Fifty micrograms of recombinant gp140 trimeric protein were delivered intramuscularly by needle injection with 20% Adjuplex vol/vol (Sigma, St-Louis, MO) as adjuvant. Blood was collected every 2 weeks after the first immunization; serum was separated and stored at −20°C until assays were performed.

### Non-human Primate Immunizations

Twelve adult male *M. mulatta* (rhesus macaques) were assigned to two experimental groups of six NHPs which were balanced for age and body weight and pair-housed at the Oregon National Primate Research Center (ONPRC) in Beaverton, OR. All animals were free of Cercopithicine herpesvirus 1, D-type simian retrovirus, simian T-lymphotrophic virus type 1, and SIV infection at the start of the study. All procedures were performed according to rules and protocols approved by the OHSU West Campus Institutional Animal Care and Use Committee.

NHPs (six per group) were co-immunized with DNA and protein at weeks 0, 4, 12, 20, 32, and 40. A total of 36 μg DNA was delivered epidermally by PMED gene gun XR-1 device (PowderMed, Oxford, UK), at a pressure of 500 psi, in 18 immunizations of 2 μg DNA each given in two non-overlapping sites along the shaved abdomen from chest to upper inner thighs. Fifty micrograms of recombinant gp140 trimeric protein were delivered intramuscularly by needle injection in the quadricep with 20% Adjuplex vol/vol (Sigma, St-Louis, MO) as adjuvant. Blood was collected every 2 weeks after the first immunization; blood was separated and serum was stored at −20°C until assays were performed. Inguinal lymph node biopsies were performed 2 weeks after the third, and fourth immunizations and 3 weeks after the last immunization.

### Phylogenetic Analyses

Unique nucleotide sequences were aligned to clade C envelope reference ZA_04_SK164B1 (accession # AY772699) with HIVAlign (http://www.hiv.lanl.gov/content/sequence/VIRALIGN/viralign.html) and manually edited in Geneious to remove indels. The DIVEIN program (http://indra.mullins.microbiol.washington.edu/DIVEIN/) was used to build maximum-likelihood (ML) phylogenetic trees rooted on the ZA_04_SK164B1 sequence using the HKY85 model. The ML trees were then visualized with the Figtree program (68).

### Motif Optimization

Envelope genes selected as vaccine candidates were motif-optimized (MO) using the Robins–Krasnitz algorithm as previously described (69, 70).

### Envelope DNA Synthesis and Cloning

The motif-optimized envelope nucleotide sequences were synthesized and cloned into the pUCminusMCS cloning vector by Blue Heron Biotechnology (Bothell, WA). The 2.5 kb envelope fragment was digested with NheI and MluI enzymes (New England Biolabs, Ipswich, MA) and gel extracted from the pUCminusMCS cloning vector before ligation to the SAP-treated pEMC^*^ expression vector with a Roche rapid DNA ligation kit (Roche Diagnostics, Indianapolis, IN). MAX Efficiency Stbl2-competent cells (Invitrogen, Carlsbad, CA) were transformed and grown at 30°C for 24 h. Clonal populations were screened by colony PCR. Positive colonies were grown in small liquid cultures at 30°C for 24 h and glycerol stocks and plasmid minipreps (Promega, Madison, WI) were generated.

### Plasmid DNA Immunogens

DNA was precipitated onto 1 μm diameter gold beads, and bullets were prepared as described (28) according to the manufacturer's instructions (Bio-Rad, Hercules, CA). For the rabbit immunogenicity study, each bullet was loaded with 2 μg total DNA with a GM-CSF to env DNA ratio of 1:10. For the macaque immunogenicity study, each bullet was loaded with 2 μg total env DNA amount. To verify that the bullets were functional, COS-7 cells were transfected with the DNA carried by the gold beads and assessed for Env protein expression (28).

### Recombinant gp140 Proteins

The gp140 DNA was derived from the gp160 envelope sequence by site-directed mutagenesis (QuickChange Multi Site-Directed Mutagenesis Kit, Stratagene, La Jolla, CA) to insert the previously described mutations (28, 29) in the primary and secondary protease cleavage sites, respectively, REKR was mutated to RSKS and KAKRR was mutated to KAISS. A large-scale endotoxin-free plasmid preparation (Qiagen, Valencia, CA) was used for stable expression in 293F cells for protein production as previously described (30).

### Abs, Peptides, and Recombinant Proteins

Anti–HIV-1 mAbs used as controls in ELISA and neutralization assays, clade C Consensus gp160 Env overlapping peptide set and recombinant protein RSC3 were obtained from the National Institutes of Health (NIH) AIDS Reagent Program.

### Antigenic Characterization of Trimers

#### ELISA

The antigenic characterization of gp140 trimeric proteins was performed as follows: Immunosorp plates (Nunc, Rochester, NY) were coated overnight with gp140 trimers at 0.5 μg/mL. After the blocking step, 17 monoclonal antibodies (VRC01, HJ16, PG9, PG16, PGT145, PGT121, PGT128, 2G12, 2F5, 4E10, F240, NIH45-46, b12, b6, A32, F105, and 39F) were tested in three-fold dilutions with a starting concentration of 20 μg/mL. Horseradish peroxidase-conjugated recombinant Goat anti-human IgG-HRP (Jackson ImmunoResearch) was used as detection reagent and the assay was developed as described (28).

#### Surface Plasmon Resonance (SPR)

The characterization of gp140 trimers was performed at 25°C on a Biacore T200 using a CM5 sensor chip and HBS-P+ buffer (10 mM HEPES, 150 mM NaCl, 0.05% P20, pH = 7.4). Protein A/G (Pierce, Rockford, IL) was covalently immobilized on all flow cells at a concentration of 50 μg/mL in acetate buffer pH 4.5 using standard amine coupling to a level of 1,500–2,000 Resonance Units (RU). The 16 monoclonals (VRC01, HJ16, PG9, PG16, PGT145, PGT121, PGT128, 2G12, 2F5, 4E10, F240, NIH45-46, b12, b6, A32, and F105) were captured onto the protein A/G surface on flow cells 2–4, leaving flow cell 1 as a reference. The antibodies were non-covalently immobilized to a level of 400 ± 50 RU by flowing for 25–60 s at a concentration of 2 μg/mL. Trimeric gp140 proteins were injected over the sensor surface using single-cycle kinetics at the following concentrations: 1.23, 3.70, 11.11, 33.33, and 100 nM. The association phase was 180 s and the dissociation phase was 300 s. Samples were maintained at 15°C before injection. Regeneration of the capture complex was achieved using a 60 s pulse of 10mM glycine pH 1.7. The data were analyzed using T200 evaluation software, with all data being double reference subtracted and normalized to the level of captured antibody.

### Binding Analyses of Polyclonal Antibodies

#### ELISA

The binding antibody response to gp140 trimeric envelope proteins was measured by ELISA as described (71).

#### SPR

Antibody affinity in rabbit sera to heterologous trimer SF612 were determined on a Biacore T200 (GE, Healthcare, Piscataway, NJ) as previously described (28).

### Peptide Scanning ELISAs

Peptide ELISA mapping was performed on pooled sera at week 22 for the rabbit study and at weeks 22 and 43 for the NHP study with overlapping linear 15-mer peptides for the gp160 clade C consensus sequence as described previously (71).

### gp70 V1V2 and RSC3 ELISA

The binding Ab response to gp70 (MLV)-V1V2 HIV-1/clade B/Case A2 protein (Immune Technology, New York, NY) was measured by endpoint ELISA with week 22 rabbit serum samples as described (39). The binding Ab resonse to the resurfaced gp120 recombinant core proteins RSC3 was determined with week 22 rabbit serum samples by endpoint ELISA as previously described (71).

### Pseudoviruses

Pseudoviruses were produced using the pSG3ΔEnv DNA plasmid encoding the HIV backbone and a plasmid encoding either homologous or heterologous envelope variants as described (72).

### Neutralization Assays

The TZM-bl assay was performed as previously described (73). All values were calculated with respect to virus only wells [(value for virus only minus cells only) minus (value for serum minus cells only)] divided by (value for virus minus cells only).

### Antibody-Dependent Cellular Cytotoxicity (ADCC) Assay

Using the assay developed by Alpert et al. (74), ADCC activity was assessed in week 22 macaque sera samples with SHIV-SF162P3-infected target cells. NKR24 cells were used as targets which were derived from CEM.NKR.CCR5 CD4+ T cells (75, 76) and obtained from the AIDS Research and Reference Reagent Program. The KHYG-1 rhCD16 effector cells were derived from the CD16-negative human NK cell line KHYG-1 (Japan Health Sciences Foundation) (77). Briefly, the NKR24 Lucase-reporter cell line is infected by spinoculation and incubated for 3–4 days before starting the assay to achieve an infection titer that is at least five-fold over background [Relative Light Units (RLU) readout of mock infected cells]. SHIV-infected NKR24 target cells are washed and combined with KHYG-1 rhCD16 cells at a ratio of effectors to targets (E:T) of 10:1 and added to the assay plate along with serially diluted sera or positive and negative mAb controls. Controls that define 100 and 0% RLU are included on the plate. The assay is incubated for 8 h after which the Lucase substrate reagent, Bright-Glo (Promega), is used to read activity as RLU indicate Lucase activity which is an indicator of cytotoxicity of infected target cells. Data are reported as the %RLU and 50% ADCC titers (78).

### Follicular Helper CD4 T Cells (TFH) Intracellular Cytokine Staining

Lymphocytes were isolated from inguinal lymph node biopsies 2 weeks after the third immunization, and CAP257 Env-specific GC T_FH_ cells were detected using a live cell Activation Induced Marker (AIM) technique described in (45) whereby T_FH_ cells were defined as CD20^**−**^CD3^+^CD4^+^PD-1^hi^CXCR5hi and Env-specific responses were measured by OX40+ and CD25+. Lymphocytes collected 3 weeks after immunizations 4 and 6 were analyzed by conventional intracellular staining where T_FH_ cells were defined as CD3^+^CD4^+^CXCR5^+^PD-1^hi^ and Env-specific responses were measured by IL-21 and IFN-γ as previously described (71).

### Statistical Analyses

#### Rabbit Study

For the *K*_dis_ evaluation, repeated measures ANOVA was used to test for examine the vaccination variation groups and time effects for the *K*_dis_. AR (1), Auto-regressive order 1, was chosen to be within subject a covariance structure. Tukey multiple comparison correction was used.

#### Macaque Study

Heterologous neutralization responses elicited by both vaccine strategies were assessed by RM 2-way ANOVA followed by Bonferroni post-test while ADCC AUC titers and T_FH_ responses were assessed by Mann–Whitney test. Correlations were determined by Spearman's test. The statistical analyses were performed either with GraphPad Prism, or SAS software packages.

## Data Availability Statement

The raw data supporting the conclusions of this article will be made available by the authors, without undue reservation, to any qualified researcher.

## Ethics Statement

The studies involving human participants were reviewed and approved by University of KwaZulu-NNatal (E013/04), the University of Cape Town (025/2004) and the University of Witwatersrand (MM040202). The patients/participants provided their written informed consent to participate in this study. The animal study was reviewed and approved by Oregon Health and Science University IACUC.

## Author Contributions

DM and AH contributed equally to this work. DM selected and designed the vaccine constructs, conceived the rabbit study, performed and analyzed experiments, prepared figures, and co-wrote the manuscript. CW isolated envelope clones. MN performed ELISA and neutralization assays. JR performed T_FH_ assays. DNS produced the gp140 proteins. DAS performed the ADCC assays. JTS analyzed the SPR data. BG and SP produced the gp160 vaccines. HR motif-optimized the vaccine sequences. BP performed statistical analyses. DF provided the PMED XR-1 gene gun device. JBS and PM assisted with data interpretation and manuscript preparation. AH conceived the macaque study, analyzed data, prepared figures, and co-wrote the manuscript. NH secured funding, supervised the conception of the studies, assisted with data interpretation, and manuscript preparation.

## Conflict of Interest

JTS was employed by GE Healthcare. The remaining authors declare that the research was conducted in the absence of any commercial or financial relationships that could be construed as a potential conflict of interest.

## Supplementary Material

Supplementary Figure 1 CAP257 Highlighter. The analysis was generated using the LANL HIV sequence database. CAP257 sequences are chronologically displayed by timepoints since infection. Nucleotide differences from the master are indicated by tic marks (red, non-silent; green, silent).

Supplementary Figure 2 Antigenic characterization of CAP257 gp140 trimers by SPR binding to anti-HIV Env monoclonal antibodies. Antibodies were captured onto protein A/G. The gp140 trimers were injected over the captured antibodies using a single-cycle kinetics method with gp140 concentrations ranging from 1.23 to 100 nM. The binding responses (expressed as resonance units, RU) are adjusted to the antibody capture level. Monoclonal antibodies are displayed based on the intensity of the measured SPR response.

Supplementary Figure 3 Longitudinal heterologous neutralizing antibodies elicited by CAP257 vaccine strategies in rabbits. Neutralization of a panel of recombinant heterologous viruses in the TZM-bl assay. **(A)** Heatmap styled diagram of neutralization titers (ID_50_) derived from rabbit serum samples after the second (week 6), third (week 14), and fourth immunization (week 22). **(B)** SF162-specific longitudinal binding and neutralizing antibodies in rabbits. Titers from 54 wpi and the 93 wpi vaccine strategies were higher compared to the 7–30 wpi strategy (*P* = 0.0049 and 0.0213, respectively). Neutralization data are expressed as ID_50_, serum dilution that neutralized 50% of the infecting virus. A decrease in RLU from a serum dilution <50 was considered as non-specific cell death and no neutralization.

Supplementary Figure 4 Durable Env-specific binding antibody responses at 2 years post-immunization. Binding antibody titers in serum from animal 24,383 to autologous trimers 54wpi_D and 93wpi_F12 and to heterologous trimer SF162 were determined by ELISA.

Supplementary Figure 5 Pre-immune sera at week 0 and sera collected 2 weeks following immunizations 4 (week 22) and 6 (week 43) were tested for binding in ELISA to **(A)** V1V2(ZM53)-2F5K and **(B)** the resurfaced core gp120 protein, RSC3 (44). Data represents midpoint titers (EC_50_) of macaque antibodies targeting conformational and linear V1V2 epitopes (42) and the CD4 binding site.

Supplementary Figure 6 Longitudinal heterologous neutralizing antibodies elicited by CAP257 vaccine strategies in NHPs. **(A)** Macaque serum samples after the second through sixth immunizations were tested for neutralization of a panel of Tier 1 and Tier 2 clade **(A–C)**, and one recombinant heterologous viruses in the TZM-bl assay. Neutralization data are expressed as ID_50_, serum dilution that neutralized 50% of the infecting virus. A decrease in RLU from a serum dilution <50 was considered as non-specific cell death and no neutralization. The positive control for each assay was a human monoclonal bNAb, and the negative control was naïve macaque plasma.
