# Supplementary figures and images for: Rapid Induction of Multifunctional Antibodies in Rabbits and Macaques by Clade C HIV-1 CAP257 Envelopes Circulating During Epitope-Specific Neutralization Breadth Development
Source: Front Immunol. 2020 Jun 2;11:984. doi: 10.3389/fimmu.2020.00984 (PMC7280454; doi:10.3389/fimmu.2020.00984)

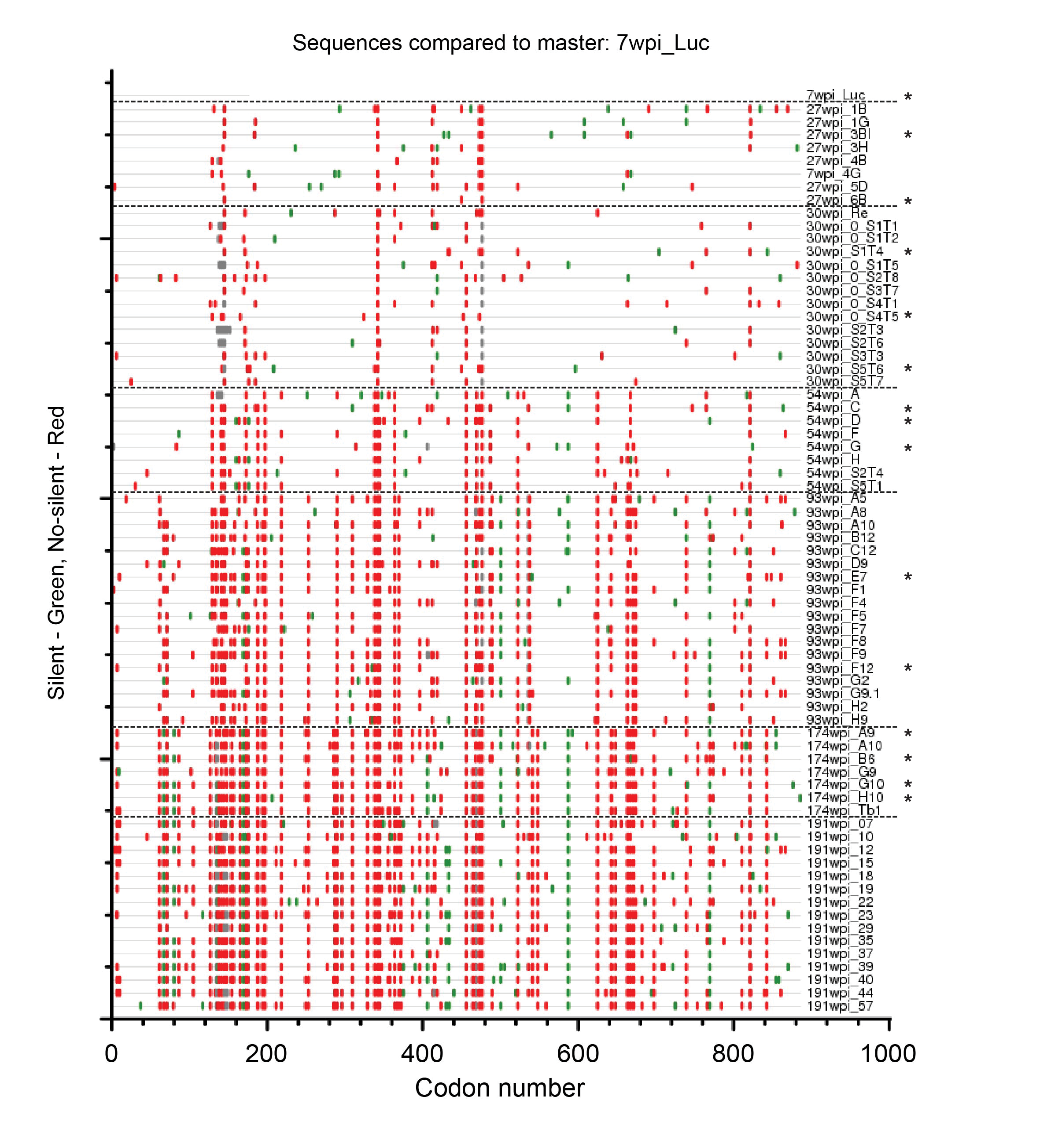

Supplement: Supplementary Figure 1 — CAP257 Highlighter. The analysis was generated using the LANL HIV sequence database. CAP257 sequences are chronologically displayed by timepoints since infection. Nucleotide differences from the master are indicated by tic marks (red, non-silent; green, silent). [file Image_1.TIF]

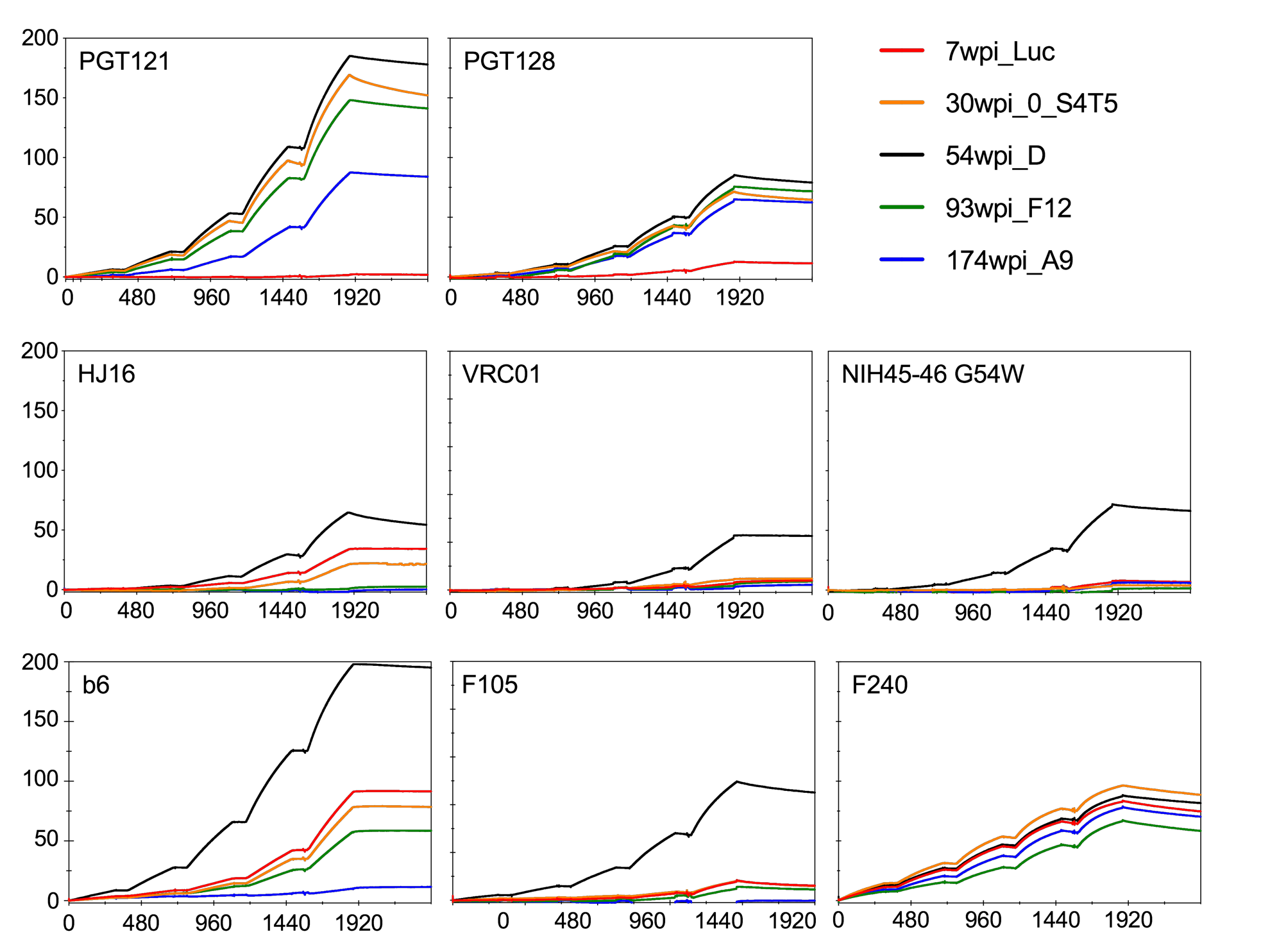

Supplement: Supplementary Figure 2 — Antigenic characterization of CAP257 gp140 trimers by SPR binding to anti-HIV Env monoclonal antibodies. Antibodies were captured onto protein A/G. The gp140 trimers were injected over the captured antibodies using a single-cycle kinetics method with gp140 concentrations ranging from 1.23 to 100 nM. The binding responses (expressed as resonance units, RU) are adjusted to the antibody capture level. Monoclonal antibodies are displayed based on the intensity of the measured SPR response. [file Image_2.TIF]

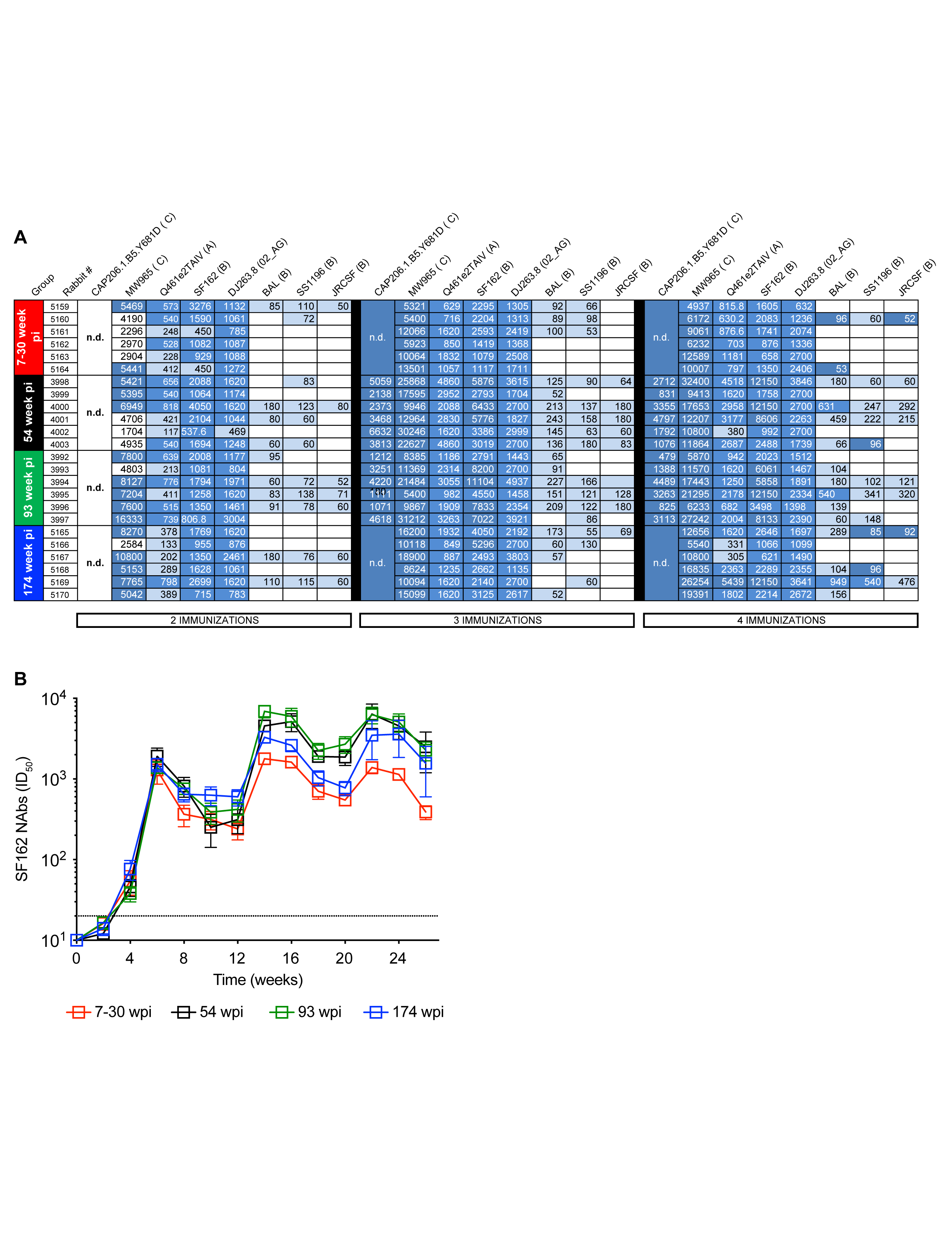

Supplement: Supplementary Figure 3 — Longitudinal heterologous neutralizing antibodies elicited by CAP257 vaccine strategies in rabbits. Neutralization of a panel of recombinant heterologous viruses in the TZM-bl assay. (A) Heatmap styled diagram of neutralization titers (ID50) derived from rabbit serum samples after the second (week 6), third (week 14), and fourth immunization (week 22). (B) SF162-specific longitudinal binding and neutralizing antibodies in rabbits. Titers from 54 wpi and the 93 wpi vaccine strategies were higher compared to the 7–30 wpi strategy (P = 0.0049 and 0.0213, respectively). Neutralization data are expressed as ID50, serum dilution that neutralized 50% of the infecting virus. A decrease in RLU from a serum dilution <50 was considered as non-specific cell death and no neutralization. [file Image_3.TIF]

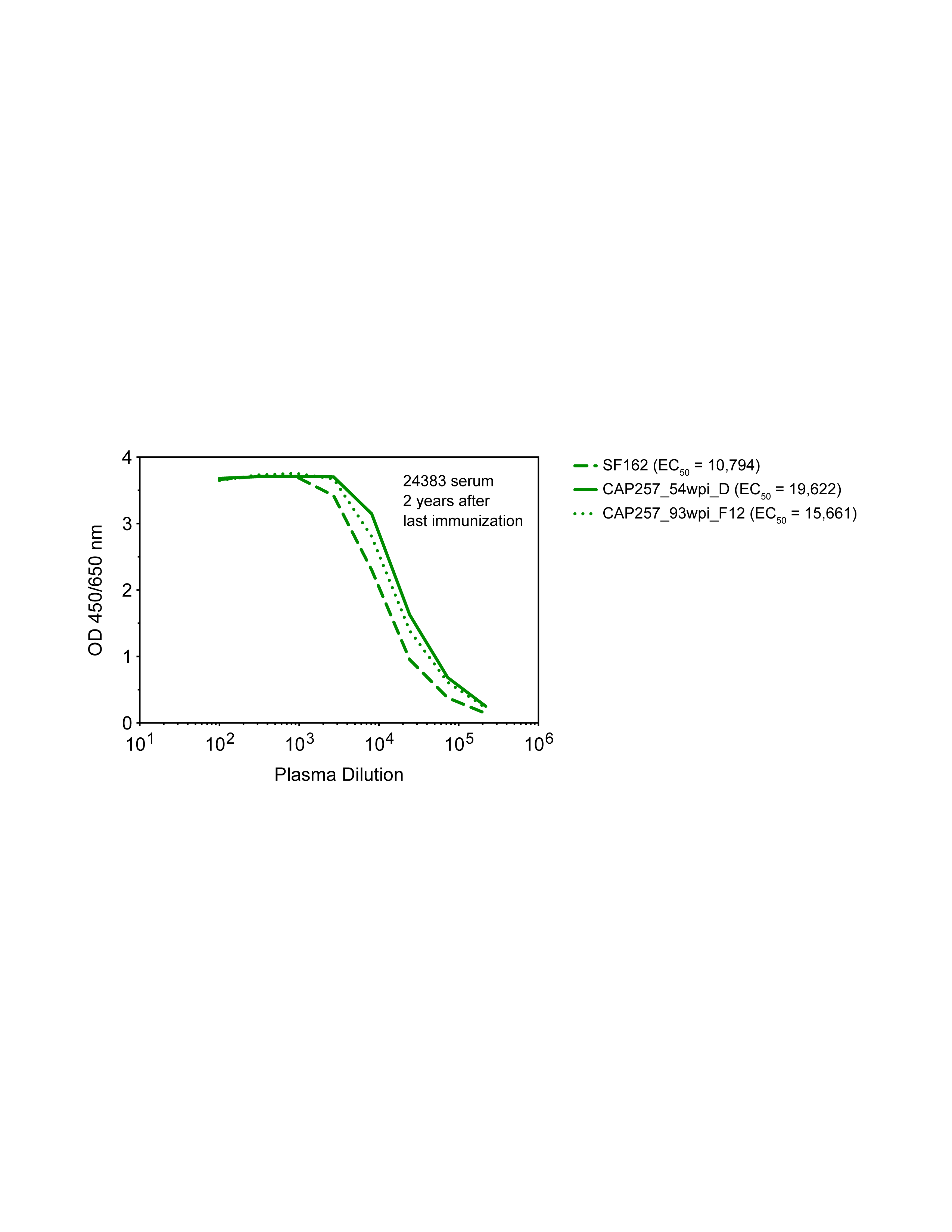

Supplement: Supplementary Figure 4 — Durable Env-specific binding antibody responses at 2 years post-immunization. Binding antibody titers in serum from animal 24,383 to autologous trimers 54wpi_D and 93wpi_F12 and to heterologous trimer SF162 were determined by ELISA. [file Image_4.TIF]

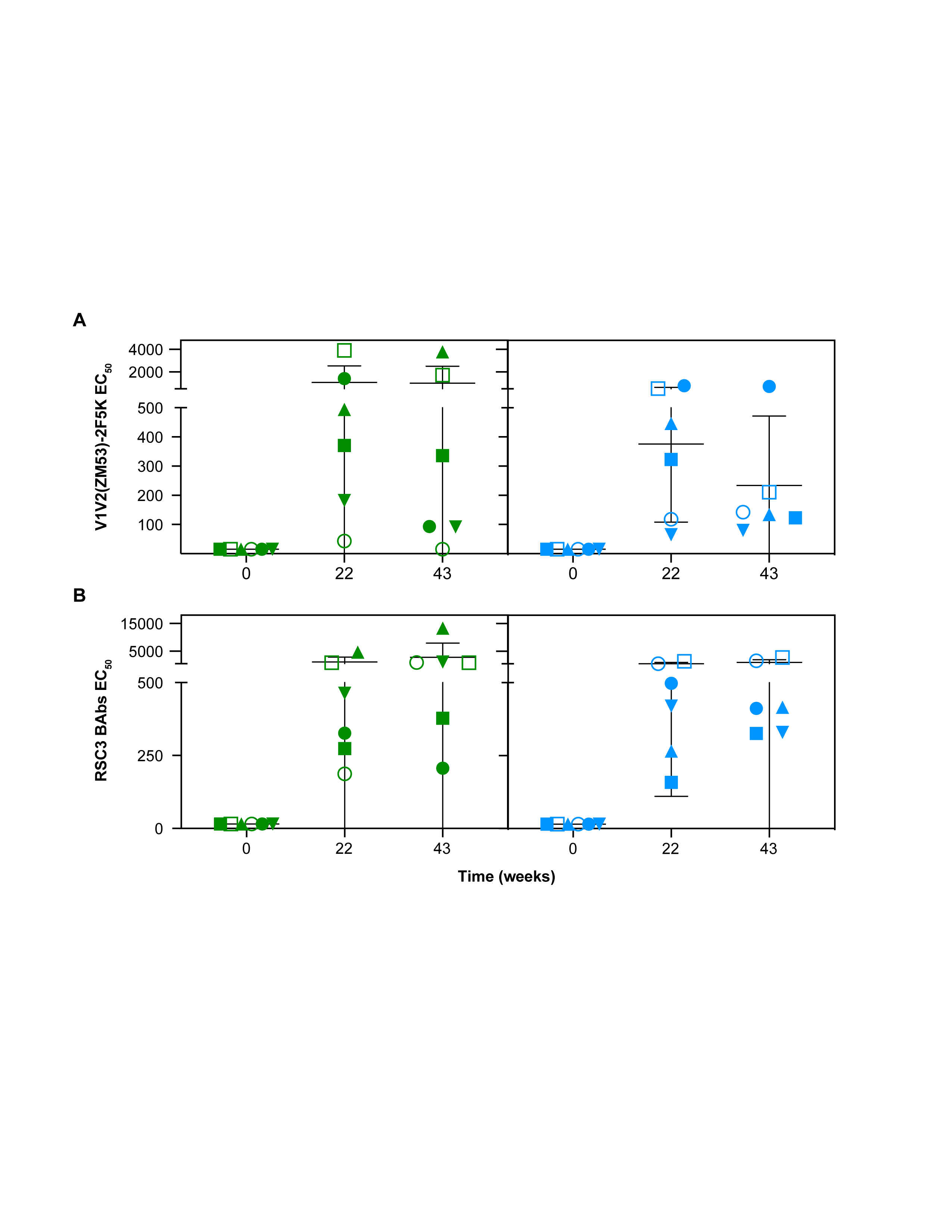

Supplement: Supplementary Figure 5 — Pre-immune sera at week 0 and sera collected 2 weeks following immunizations 4 (week 22) and 6 (week 43) were tested for binding in ELISA to (A) V1V2(ZM53)-2F5K and (B) the resurfaced core gp120 protein, RSC3 (44). Data represents midpoint titers (EC50) of macaque antibodies targeting conformational and linear V1V2 epitopes (42) and the CD4 binding site. [file Image_5.TIF]

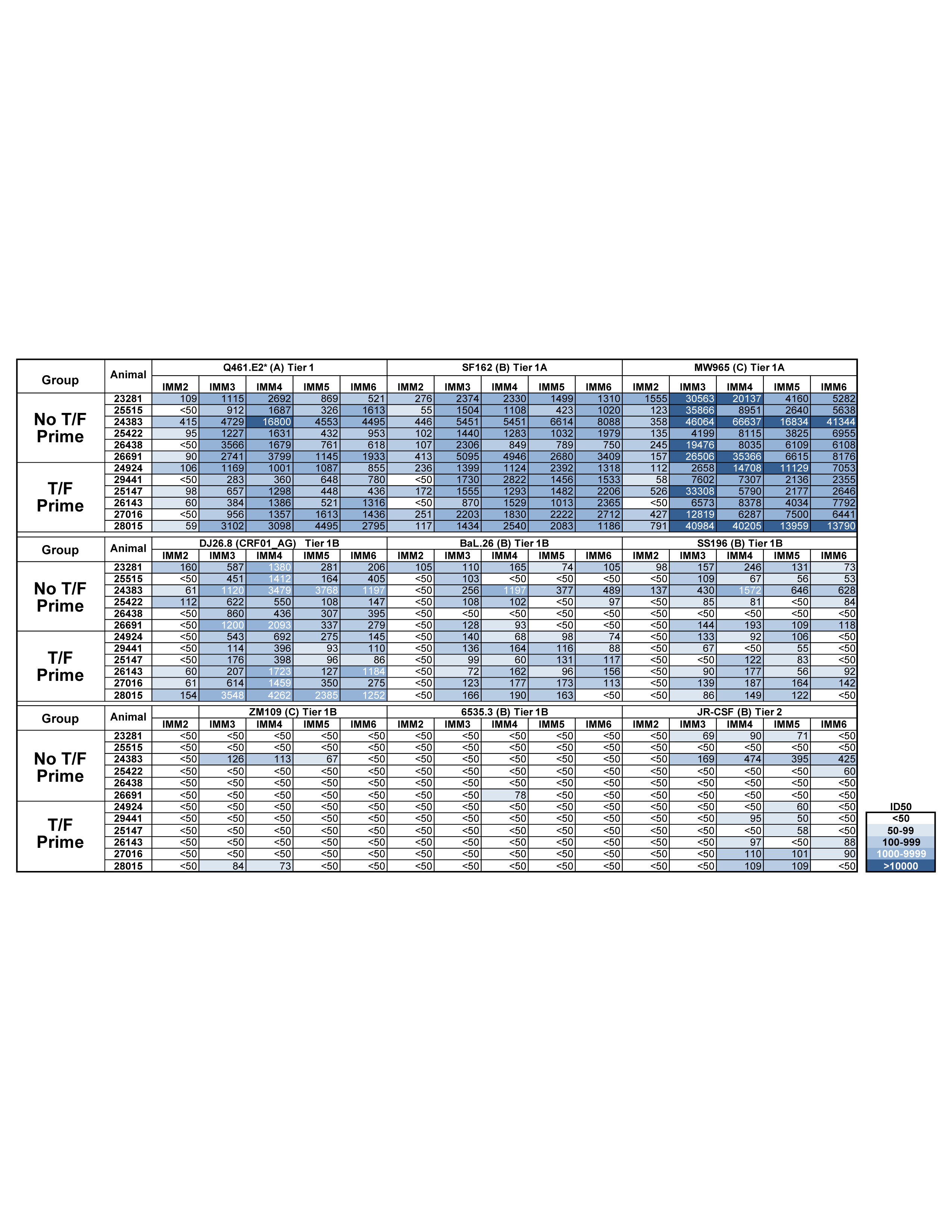

Supplement: Supplementary Figure 6 — Longitudinal heterologous neutralizing antibodies elicited by CAP257 vaccine strategies in NHPs. (A) Macaque serum samples after the second through sixth immunizations were tested for neutralization of a panel of Tier 1 and Tier 2 clade (A–C), and one recombinant heterologous viruses in the TZM-bl assay. Neutralization data are expressed as ID50, serum dilution that neutralized 50% of the infecting virus. A decrease in RLU from a serum dilution <50 was considered as non-specific cell death and no neutralization. The positive control for each assay was a human monoclonal bNAb, and the negative control was naïve macaque plasma. [file Image_6.TIF]
